# Transcriptionally dynamic progenitor populations organised around a stable niche drive axial patterning

**DOI:** 10.1242/dev.168161

**Published:** 2019-01-02

**Authors:** Filip J. Wymeersch, Stavroula Skylaki, Yali Huang, Julia A. Watson, Constantinos Economou, Carylyn Marek-Johnston, Simon R. Tomlinson, Valerie Wilson

**Affiliations:** 1 MRC Centre for Regenerative Medicine, Institute for Stem Cell Research, School of Biological Sciences, University of Edinburgh, 5 Little France Drive, Edinburgh EH16 4UU, UK; 2 RIKEN Center for Biosystems Dynamics Research, 2-2-3 Minatojima-minamimachi, Chuo-ku, Kobe, Hyogo 650-0047, Japan; 3 Department of Biosystems Science and Engineering, ETH Zürich, 4058 Basel, Switzerland

**Keywords:** Mouse, Neuromesodermal, Lateral and paraxial mesoderm, Notochord progenitors, Hox

## Abstract

The elongating mouse anteroposterior axis is supplied by progenitors with distinct tissue fates. It is not known whether these progenitors confer anteroposterior pattern to the embryo. We have analysed the progenitor population transcriptomes in the mouse primitive streak and tail bud throughout axial elongation. Transcriptomic signatures distinguish three known progenitor types (neuromesodermal, lateral/paraxial mesoderm and notochord progenitors; NMPs, LPMPs and NotoPs). Both NMP and LPMP transcriptomes change extensively over time. In particular, NMPs upregulate Wnt, Fgf and Notch signalling components, and many Hox genes as progenitors transit from production of the trunk to the tail and expand in number. In contrast, the transcriptome of NotoPs is stable throughout axial elongation and they are required for normal axis elongation. These results suggest that NotoPs act as a progenitor niche whereas anteroposterior patterning originates within NMPs and LPMPs.

## Abstract (precis)

**Summary:** During anteroposterior axis elongation, neuromesodermal and lateral mesoderm axial progenitors undergo dynamic transcriptomic change, while the adjacent notochord progenitors are stable and coordinate elongation.

## INTRODUCTION

The anteroposterior axis of the vertebrate embryo emerges in a head-to-tail sequence from a growth zone termed the primitive streak in early embryos, and the tail bud in later embryos. This region, located at the posterior tip of the embryo, continuously produces the spinal cord, notochord, paraxial and lateral/ventral mesoderm over the anteroposterior axis (reviewed by Henrique et al., 2015; Wilson et al., 2009). The progenitors of spinal cord and paraxial mesoderm (neuromesodermal progenitors or NMPs) arise just before the start of somitogenesis, and are maintained in the caudal lateral epiblast (CLE) and node-streak border (NSB) of the primitive streak region and the chordoneural hinge (CNH) of the tail bud until elongation ceases at around 65 somite pairs (s) in the mouse. Population and clonal analyses indicate that these cells behave as a stem cell population (Cambray and Wilson, 2007; Tzouanacou et al., 2009). Specifically, they can produce progenitors that remain in the primitive streak and tail bud, as well as differentiated paraxial mesoderm or neurectoderm. Furthermore, NMPs transplanted from late to early embryos can reset their identity to produce more anterior segments of the axis (Cambray and Wilson, 2002; McGrew et al., 2008). Despite this functional stem cell-like behaviour, there are intriguing temporal changes in gene expression throughout the progenitor region, likely including NMPs (Cambray and Wilson, 2007; Gomez et al., 2008; Olivera-Martinez et al., 2012), and a recent study comparing individual NMPs with their descendant mesoderm at two developmental stages shows that some temporal differences occur in NMPs themselves (Gouti et al., 2017). This suggests that, similar to other ‘stem cells’ in the embryo that produce distinct differentiated phenotypes over time, such as neural or neural crest stem cells (Temple, 2001; White et al., 2001), NMPs do not strictly self-renew. It is thus unknown whether NMPs, which act as stem cells, in fact contain temporal anteroposterior patterning information.

A number of studies have highlighted a crucial role for Hox genes in anteroposterior axial patterning (reviewed by Deschamps and van Nes, 2005; Mallo et al., 2009). The sequential activation of Hox genes from paralogous group (PG) 1 to PG13 in any of the four vertebrate clusters (HoxA-D), depending on their position within the cluster, is a canonical property of Hox genes, termed temporal collinearity. Temporal collinearity has been demonstrated *in vitro* (Lippmann et al., 2015), and for a minority of mouse Hox genes *in vivo* (Izpisua-Belmonte et al., 1991; Soshnikova and Duboule, 2009; Tschopp et al., 2009). For those few that have been studied in detail, activation begins at the posterior primitive streak and spreads anteriorly (Forlani et al., 2003; Iimura and Pourquié, 2006). However, the spatiotemporal expression of most Hox genes in the mouse progenitor region, and specifically their expression in known progenitor types, remains unclear.

Two further populations of axial progenitors have been described, but characterised in less detail. Notochordal progenitors, which we term here ‘NotoPs’, are also retained for relatively long periods during axial elongation, in the ventral layer of the node at the anterior end of the primitive streak (Beddington, 1994; Wilson and Beddington, 1996). Although a comprehensive temporal fate map of the lateral plate mesoderm (LPM) has not been reported, fate maps at individual stages show that anterior and trunk LPM progenitors (LPMPs) are present in the primitive streak prior to somitogenesis (Castillo et al., 2016; Kinder et al., 1999; Smith et al., 1994; Taguchi et al., 2014). The posteriormost LPM, forming the peri-cloacal mesenchyme, is derived from LPMPs in the early somite-stage primitive streak (Cambray and Wilson, 2007; Wymeersch et al., 2016). The LPM (and potentially also its progenitors) provides important signals that regulate the transition from production of the trunk to the tail. Interestingly, signalling from NMPs to LPMPs may be important for sustaining and patterning the LPMP population (Aires et al., 2016; Jurberg et al., 2013). Aside from this interaction, however, the interplay between different progenitors as axis elongation proceeds is not clear.

Thus, despite an accumulating body of information, it still remains obscure: (1) when, and in which cells, do known patterning events, such as Hox acquisition, take place; and (2) what interactions occur between different axial progenitor populations?

To answer these questions, we determined the spatiotemporal transcriptome for axial progenitors throughout axis elongation. We find that NMPs, LPMPs and NotoPs show distinct expression profiles, whereas NMPs are similar to their immediate mesoderm-committed descendants. Furthermore, we show transcriptional changes occur in LPMPs and NMPs over time; in the latter, the major change occurs between early somitogenesis and completion of trunk morphogenesis. We also present evidence that NotoPs are a stable integrator of the behaviour of these progenitors.

## RESULTS

### Temporal differences in transcriptome predominate over progenitor identity

**Fig. 1.**
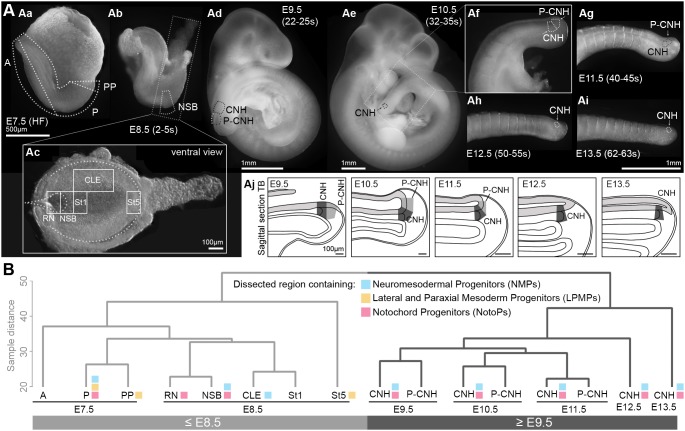
**Microarray analysis of primitive streak and tail bud regions.** (A) Schema of dissected embryonic regions. (Aa) In headfold (HF) stage embryos, three regions were isolated: A, anterior; P, posterior; PP, proximal posterior. (Ab) At E8.5 (2-5 s), five regions were isolated as shown in Ac: RN, rostral node; NSB, node-streak-border; St1, rostral 1/5 of the streak; St5, caudalmost 1/5 of the streak; CLE, caudal lateral epiblast beside the rostral 3/5 length of the streak. (Ad-i) The chordoneural hinge (CNH) was dissected in E9.5-E13.5 tail buds. The region just posterior to the CNH (P-CNH) was dissected in E9.5-E11.5 embryos. (Aj) Schema of tail bud (TB) sections with the dissected domains. (B) Unsupervised hierarchical clustering of all samples. Coloured squares highlight the progenitors present in the samples: NMPs (blue), LPMPs (yellow) and NotoPs (pink).

We collected RNA of microdissected embryonic regions according to Fig. 1A, and performed Illumina microarray hybridisation detecting ∼45,000 transcripts. Samples corresponded to regions of known or expected differential fate in and around the primitive streak and tail bud (Table 1; Fig. 1; Materials and Methods). Importantly, although each sample contained a mixture of cell types (Fig. S1A), comparison of samples containing target and non-target cell types allowed us to extract gene expression signatures in cell types of interest. To validate the accuracy and reproducibility of dissection, we performed qRT-PCR on a series of markers with known regional expression on independently dissected regions of the primitive streak (Fig. S1B). We also performed *in situ* hybridisation on primitive streak/tail buds from E9.5-13.5 for known markers (Fig. S2). These analyses showed, in all cases examined, similarity between the intensity values in the microarray analysis and the corresponding independently validated measurements.

**Table 1.**
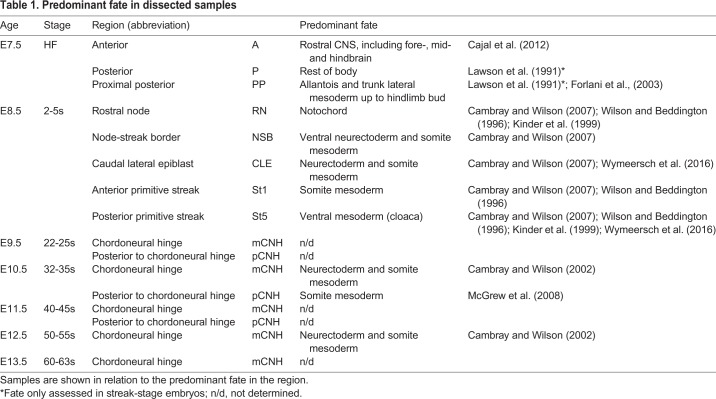
**Predominant fate in dissected samples**

We performed unsupervised hierarchical clustering on all samples, expecting separation into fate-based clusters: NMP-containing (NSB, CLE and CNH), exclusively mesoderm-fated NMP descendants (St1, P-CNH) and LPMP-containing regions (E7.5 PP and E8.5 St5). Unexpectedly, NMP regions were more related to their contemporary non-NMP neighbours than to NMP-containing samples at other stages. This suggests that NMPs are not highly transcriptionally divergent from their immediate mesoderm-committed descendants in the primitive streak and tail bud, and that, instead, embryonic age constitutes a distinct transcriptomic signature in NMPs and their descendants. Moreover, samples formed two major clusters: an early group corresponding to stages up to E8.5 and a late group composed of samples from E9.5 onwards. Within the ‘early’ grouping, the transcriptome of St5 was most divergent from other samples (Fig. 1B).

### Analysis of differential expression at E8.5 reveals spatial domains corresponding to progenitor subpopulations

**Fig. 2.**
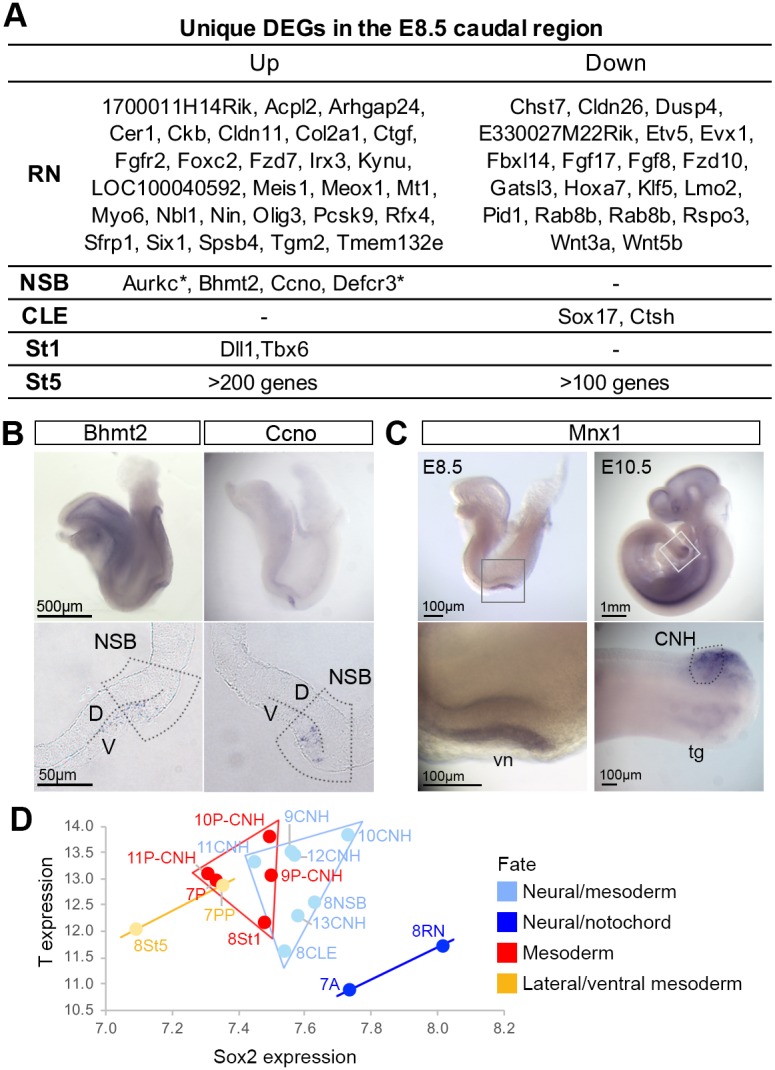
**Spatial analysis of the primitive streak region.** (A) Unique DEGs to each E8.5 region (≥1.5-fold change compared with other samples; for unique DEGs in St5, see Table S1). Asterisk indicates that no specific *in situ* probe could be constructed. (B) *Bhmt2* and *Ccno* expression in the ventral (V) but not dorsal (D) NSB layer. (C) Whole-mount *in situ* hybridisation for *Mnx1*. tg, tail gut; vn, ventral node. (D) *Sox2* versus *T* intensity values for all samples. Abbreviations are preceded by embryo age; colours indicates fate. Regions of similar fate are enclosed by a coloured line.

To investigate transcriptomic differences in known fated regions at E8.5, we first analysed differentially expressed genes (DEGs; fold change (FC)≥1.5, *P*≤0.05) between different E8.5 samples (Fig. 2A). Consistent with the clustering analysis, St5 was most divergent from the other E8.5 samples, with over 300 unique DEGs. Upregulated genes included expected markers of the posterior streak (e.g. *Bmp4*, *Cdh5*, *Flt1*, *Hhex*, *Tbx3* and *Tbx4*) (Fong et al., 1996; Fujiwara et al., 2002; Naiche et al., 2011; Scialdone et al., 2016; Thomas et al., 1998). Genes regarded as markers of the primitive streak excluding node and notochord (e.g. *Fgf8*, *Fgf17*, *Wnt3a* and *Wnt5b*) were depleted in St5, underlining its distinct character from the rest of the streak (Cambray and Wilson, 2007; Maruoka et al., 1998; Takada et al., 1994). Genes specifically upregulated in the RN included markers of emergent notochord, such as *Cerl* (Belo et al., 1997). Markers of neural (e.g. *Olig3*) and somite (e.g. *Meox1)* differentiation were also enriched, consistent with the inclusion of emerging neurectoderm and incipient somites as minor cell populations expected in this sample (Fig. S1A). Two endoderm markers, *Sox17* and *Ctsh* (Chen et al., 2013; Kanai-Azuma et al., 2002), were specifically downregulated in the CLE, consistent with the dissection of endoderm away from the CLE. *Tbx6*, a known marker of the primitive streak midline and paraxial mesoderm determinant, and *Dll1*, a known target of *Tbx6* (White and Chapman, 2005), were the only genes showing enrichment in St1, reflecting the paraxial mesoderm fate of this region (Cambray and Wilson, 2007).

The above data provide broad validation of the microarray datasets and suggest that they are appropriate for a search for uniquely enriched transcripts in E8.5 NMPs. Nevertheless, no uniquely upregulated genes were detected in the CLE. Only four genes were upregulated specifically in the NSB. *In situ* hybridisation for those for which unique probes could be designed, *Bhmt2* and *Ccno*, confirmed their specific localisation in the NSB (Fig. 2B). However, their expression was confined to the ventral layer corresponding to the crown of the node, rather than to the NMP-containing dorsal layer. The levels of known NMP markers *T* and *Sox2* correlated well with protein levels measured by immunofluorescence, underlining the accuracy of dissection (Wymeersch et al., 2016) (Fig. 2D). Specifically, levels of *Sox2* correlated with neural fate in the sample, whereas levels of *T* reflected its high expression in the notochord and posterior streak, as well as anterior streak midline (Wymeersch et al., 2016). However, we found no significantly upregulated transcripts in both NSB and CLE, the two NMP-containing areas. Last, we examined the expression of another candidate NMP marker, *Mnx1* (Harrison et al., 1999) (Fig. 2C), which was expressed in the ventral node region at E8.5, but expressed in the E10.5 CNH, making it a potential late NMP marker. Thus, despite the identification of genes with both known and novel differential expression in LPMPs and NotoPs, our analysis did not identify single genes specifically enriched in all NMP-containing areas, either due to a lack of unique markers or because these were below threshold detection levels.

**Fig. 3.**
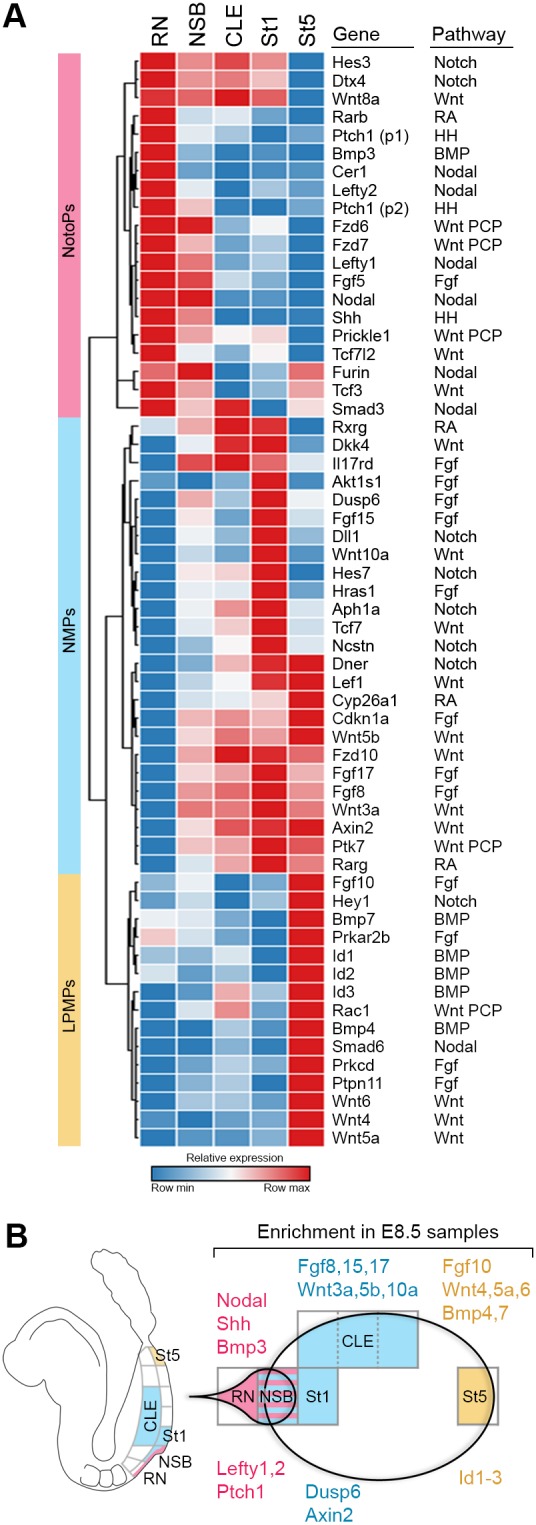
**Spatial domains correspond to progenitor subpopulations.** (A) Hierarchical clustering of selected KEGG pathway components reveals three broad domains of signalling (≥1.5-fold change across E8.5 samples), in accordance with the progenitors they contain (NotoPs, NMPs or LPMPs). (B) Schema showing enrichment of signalling ligands and their transcriptional targets in the primitive streak region.

The regional expression of signalling molecules in the analysis above led us to systematically examine the spatial localisation of signalling pathway activity at E8.5. We analysed the Kyoto Encyclopedia of Genes and Genomes (KEGG; Kanehisa et al., 2016) components of the Wnt, Notch, retinoic acid (RA), Nodal, Hedgehog and BMP signalling pathways, which are active in the primitive streak and also involved in axial patterning (reviewed by Wilson et al., 2009). Hierarchical clustering of DEGs belonging to these signalling pathways (≥1.5FC across E8.5 samples) showed three broad domains where signalling molecules were expressed (Fig. 3A): (1) the RN and NSB; (2) the NSB, CLE and St1; and (3) St5. The RN-NSB domain was uniquely characterised by Shh pathway member upregulation, whereas the NSB-CLE-St1 domain showed upregulation of Notch pathway members. *Bmp4* and *Bmp7* were uniquely upregulated in St5. In addition to these unique pathway components, several Wnt, Fgf, RA and Nodal components were expressed in more than one region. However, the enrichment of individual pathway members respected these domains. Moreover, the Nodal response genes *Lefty1* and *Lefty2*, and the Hedgehog target *Ptch1* were upregulated in the RN-NSB domain, whereas Fgf and Wnt target genes (*Dusp6* and *Axin2* respectively) were upregulated in NSB-CLE-St1. Bmp targets *Id1*, *Id2* and *Id3* were upregulated in St5, indicating that these signalling pathways have localised activation patterns (Fig. 3B).

To determine whether these patterns were specific to signalling pathways known to operate in primitive streak morphogenesis, or reflected more global patterns of gene expression, we analysed all DEGs at E8.5 via hierarchical clustering and explored their function in the STRING online database (Franceschini et al., 2013) (Fig. S3A). This expanded analysis also grouped genes into the three major categories identified above, indicating that these domains contain cells with broadly similar transcriptomes. Interestingly, in the NSB-CLE-St1 cluster, we identified five genes in addition to *Dll1* with modest (<1.5x) upregulation in the midline streak relative to the CLE: *Aph1a*, *Ncstn*, *Ctbp2*, *Dvl1* and *Kat2a*, which are also associated with Notch signalling (KEGGID:04330). In the posterior primitive streak, we observed additional upregulated ligands and receptors involved in vascular development (GO.0072358), including *Pdgfra*, *Adora2b*, *Tgfb1*, *Bmp7*, *Fgf10*, *Efna1*, *Cxcl12*, *Wnt4/5b/6* and *Vegfa*, consistent with the lateral/ventral mesoderm origin of blood vessels (Fig. S3B; Table S2).

Thus, three distinct transcriptomic signatures characterise the E8.5 primitive streak region domains corresponding to the three known progenitor populations: RN-NSB (NotoPs), NSB-CLE-St1 (NMPs and their descendants) and St5 (LPMPs). These data further suggest that the three progenitor types respond to differing signalling pathways that are already known to be functional in axis development. Furthermore, the inclusion of the CLE and midline primitive streak (St1) in a single domain reflects the progression of NMPs towards mesoderm commitment. Intriguingly, it suggests that transcriptional change during this process is minor, although Notch signalling component transcription may increase along with mesoderm commitment.

### The LPMP population undergoes temporal change

**Fig. 4.**
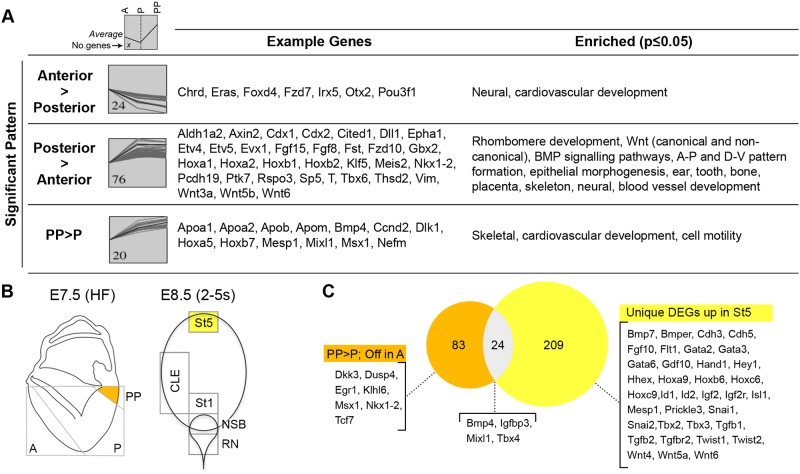
**Temporal transcriptomic changes in LPMPs.** (A) Significant patterns at E7.5 and enriched terms (*P*≤0.05, defined by permutation test in STEM). Grey boxes show the number of genes in each pattern; the black line indicates their average. (B) Schematic diagram of headfold stage embryo (E7.5 HF) and E8.5 primitive streak area with LPMP-containing regions coloured in yellow. (C) Genes uniquely upregulated in E7.5 PP versus P (orange) and DEGs unique to E8.5 St5 (yellow) outnumber common genes (grey).

To examine temporal changes between E7.5-E8.5 LPMPs, we first examined the E7.5 transcriptome. Short Time-series Expression Miner (STEM) analysis (Ernst and Bar-Joseph, 2006) indicated several significantly enriched profiles. Expected enrichment of primitive streak-specific markers (e.g. *Evx1*, *Fgf8*, *T*) (Fig. 4A; Table S3) in the posterior region and neural/emergent notochord markers anteriorly (e.g. *Otx2*, *Pou3f1*, *Chrd* and *Foxd4*) (Iwafuchi-Doi et al., 2012; Tamplin et al., 2008) provided confidence in the validity of these E7.5 samples. In the proximal-posterior region containing LPMPs, known posterior markers, including *Bmp4*, *Mesp1* and *Mixl1* (Arnold and Robertson, 2009), were enriched. Thus, transcriptome differences at E7.5 agree well with previously reported expression patterns, and suggest that LPMPs represent a transcriptionally distinct region at E7.5.

We compared 97 genes specifically upregulated in the proximal posterior region at E7.5 with the 233 genes upregulated in St5 at E8.5. (Fig. 4B,C; Fig. S4; Table S4). A relatively small overlap between these sets (*n*=24) included known markers of the posterior primitive streak, including *Bmp4*, *Mixl1*, *Tbx4* and *Efna1* (Chapman et al., 1996; Duffy et al., 2006; Lawson et al., 1999; Pearce and Evans, 1999). Enriched GO terms for this overlapping subset of genes included blood vessel morphogenesis and regulation of epithelial cell migration (*Ctsh*, *Tbx4*, *Etv2*, *Ets1*, *Bmp4* and *Efna1*) (GO:0001568 and GO:0010632). Thus, the E7.5 PP and E8.5 St5 regions, which contain prospective lateral mesoderm, show prominent differential gene expression with a subset of shared gene expression that may be useful as markers of LPMPs.

### Temporal changes in NMPs

**Fig. 5.**
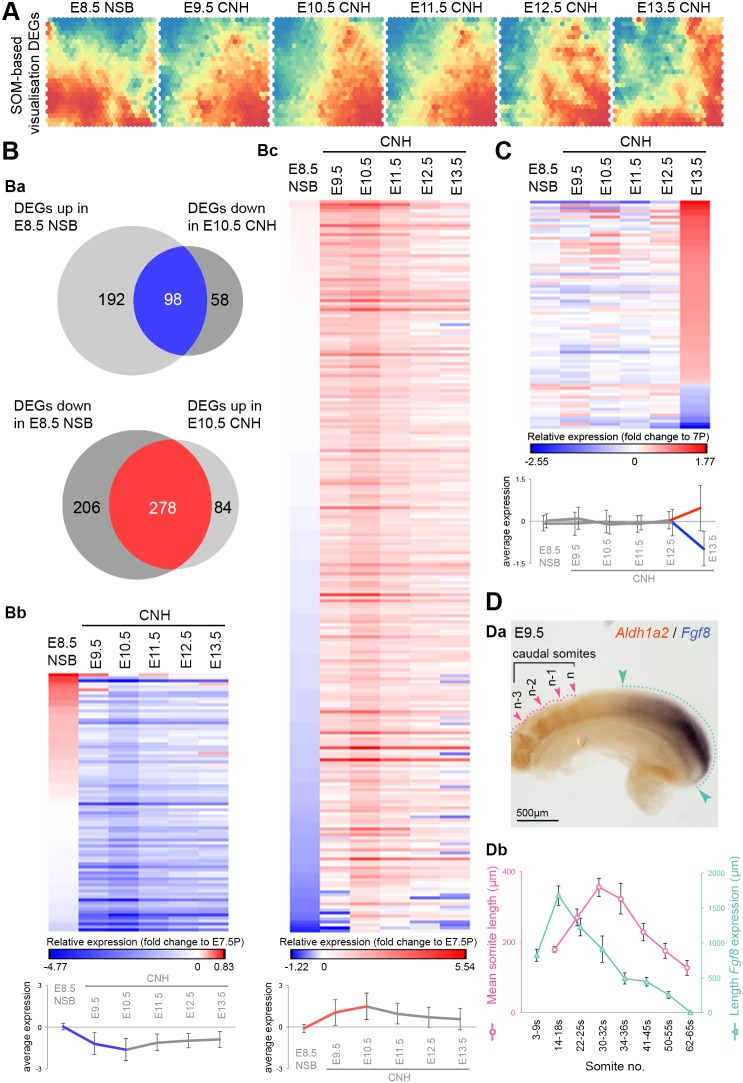
**Transcriptomic changes in NMPs during primitive streak-to-tail bud transition.** (A) Self-organising map (SOM)-based visualisation of DEGs. (Ba) Venn diagrams with upregulated DEGs in the E8.5 NSB versus those downregulated in the E10.5 CNH and vice versa (≥1.5-fold change across NMP-containing samples). Expression heatmaps of downregulated (Bb) and upregulated DEGs (Bc) in NMP-containing regions. (C) DEGs in the E13.5 CNH. All heatmap values are relative to 7P, with the mean±s.d. shown below. (Da) *In situ* hybridisation in the E9.5 tail bud for *Fgf8* and *Aldh1a2*. (Db) The anteroposterior length of *Fgf8* expression domain and the length of the four caudalmost somites are shown in relation to somite number (n) and peak at E9.5 (14-18 s) and E10.5 (30-32 s), respectively. Data are mean±s.d. (see also Fig. S8).

Comparison of NMP-containing regions with their non-NMP neighbours between all samples (up in NSB±CLE and all CNH) did not identify any enriched transcripts. Instead, the NMP transcriptome changed over developmental time (Fig. 1B). We therefore further investigated the transcriptional changes occurring specifically in topologically equivalent NMP-containing regions (the NSB and CNH) over time. Using the E7.5 posterior region as a baseline, we allowed the DEGs at each stage to form self-organising maps (Spielman and Folch, 2015), which highlight gene expression changes as a matrix of patterns and allow visualisation of periods of flux and stability in gene expression profiles (Fig. 5A). This showed that a sharp shift in gene expression occurred between E8.5 and E9.5, and a less prominent change occurred at E12.5-E13.5. We further examined DEGs uniquely up- or downregulated in any one of these samples. The majority of these were found in the E8.5 NSB and E10.5 CNH, with a smaller set at E13.5 (Table S5). Interestingly, a large proportion of the genes that were upregulated at E8.5 were downregulated at E10.5, and vice versa (Fig. 5Ba). The profiles of these genes showed a reciprocal pattern with the majority of the change occurring between E8.5 and E9.5, whereas expression returned towards E7.5 levels after E10.5 (Fig. 5Bb,Bc). Furthermore, most of the genes that did not fall in the intersections between these categories (e.g. up at E8.5 but not down at E10.5) respected the above trend, although the level of up- or downregulation was less than the 1.5-fold cut-off (Fig. S5). In contrast, the genes changing at E13.5 showed no other consistent change earlier in development (Fig. 5C). Thus, the period between E8.5 and E10.5 marks a major transition in gene expression in NMP-containing regions, with most of the shift occurring in the first 24 h of this period.

The genes undergoing transition between E8.5 and E10.5 included known markers of the primitive streak and not the notochord. Furthermore, DEGs enriched in NMPs versus nascent mesoderm in a parallel single cell analysis showed prominent temporal differences (Gouti et al., 2017). These correlated well with our list of DEGs between E8.5 and E10.5 (Fig. S5C,D), supporting the idea that the temporal changes at the NSB and CNH are specific to NMPs and not NotoPs. Genes that were downregulated between E8.5 and E10.5 included pluripotency-associated markers, e.g. *Pou5f1*, *Klf5*, *Lin28*, *Dnmt3b* and *Zscan10* (Ng and Surani, 2011; Takahashi and Yamanaka, 2006; Wang et al., 2007), and markers of the early primitive streak, such as *Cdh1*, *Cdx1*, *Cited2* and *Fst* (Albano et al., 1994; Dunwoodie et al., 1998; Malaguti et al., 2013; Meyer and Gruss, 1993). Genes that were upregulated at E10.5 included members of signalling pathways known to be expressed widely in the primitive streak and tail bud, e.g. *Wnt5a* and *Fgf8* (Crossley and Martin, 1995; Yamaguchi et al., 1999).

STEM analysis, to determine any additional temporal patterns, identified seven significantly enriched patterns (Fig. S6A; Table S3), of which two overlapped with the previously identified set of genes that were downregulated between E8.5 and E10.5 (n_genes_=160 and 42). A group of 139 genes peaked at E10.5 and overlapped with those upregulated between E8.5 and E10.5. Further groups of 46, 43 and 98 genes showed variations of this pattern with slightly broader peaks, whereas 37 genes peaked at E13.5. The lack of any other patterns indicates that changes between E8.5 and E10.5 constitute the major transcriptional diversity in NMPs over time. A further set of 46 members of transcription factor families or signalling pathways (Hox, Fox, Tbx, Pou, Wnt, Fgf, Notch and RA) was identified by correlation with the pattern of a typical profile for the 139 genes peaking at E10.5, i.e. that of *Wnt5a* (Fig. S6B; Table S6). Combining these datasets generated a list of 313 genes upregulated between E8.5 and E10.5, the expression of which declined thereafter (Fig. S6C).

### Analysis of genes upregulated between E8.5 and E10.5

A previous analysis in chick (Olivera-Martinez et al., 2014) focused on the differentiation of NMPs in the ‘stem zone’ (equivalent to the CLE) towards neural fates. The intersection of our compiled list of 313 genes upregulated at E10.5 with genes upregulated in the chick CLE versus the emerging neural tube contains 16 genes, most of which are known primitive streak markers (Fig. S7A), including Wnt pathway members and their targets (*Wnt5a*, *Wnt5b*, *Rspo3*, *T* and *Evx1*), Fgf pathway members and their targets (*Fgf8*, *Fgf18*, *Il17rd* and *Cyp26a1*), steroid signalling (*Greb1*), and epithelial-to-mesenchymal transition (*Zeb1*). Therefore, the genes upregulated at E10.5 represent a group of evolutionarily conserved primitive streak markers expressed in both NMPs and (as no marker uniquely defines NMPs) their committed NMP descendants.

GO term analysis (Fig. S6D) and manual annotation (Table S7) of this list identified genes associated with the Wnt, Fgf (Ras/Mapk/PI3K/Akt) and Notch signalling pathways, as well as the negative regulator of RA signalling *Cyp26a1*. Wnt, Fgf and Notch signalling pathways are known to be active in the primitive streak/tail bud, and are important for axial elongation, while downregulation of RA synthesis characterises the middle period of axial elongation. However, a coordinated quantitative peak in expression of these genes during trunk morphogenesis has never been reported. In addition, GO and KEGG terms associated with butanoate and steroid metabolism were also enriched. Members of several metabolic pathways were upregulated, including transcripts of the glycolytic enzymes *Eno3* and *Pgm2* recently shown to be enriched in the tail bud relative to anterior PSM, as part of a general upregulation of glycolysis (Oginuma et al., 2017). Furthermore, cell cycle regulators, extracellular matrix molecules (in particular those associated with microfibril formation) and chromatin modifiers were also upregulated in this cohort of genes (Fig. S6D; Table S7). This suggests that E9.5-E10.5 NMPs reach a maximal level of signalling, metabolic and transcriptional regulatory activity as they lay down the posterior trunk and anterior tail bud. Interestingly, the expression profile of the temporally upregulated genes correlates with an expansion in NMP numbers between E8.5 and E9.5, and a subsequent decline between E10.5 and E13.5 (Wymeersch et al., 2016). Several of the genes upregulated at E10.5 (70/313) are bound by Sox2 (13), T (17) or both transcription factors (40) in NMPs (Koch et al., 2017). Moreover, 20/313 genes were targets of β-catenin in human ES cells, and a larger subset was activated by CHIR99021-mediated Wnt/β-catenin stimulation of EpiSCs (Fig. S7) (Funa et al., 2015; Tsakiridis et al., 2014). This suggests that activation of Wnt/β-catenin signalling and its direct target T may account for some of the increase in expression of these genes. Interestingly, many β-catenin targets are also members or targets of the Fgf signalling pathway (*Fgf 8*, *Fgf17 Fgf18*, *Dusp6* and *Il17rd*) (Aulehla et al., 2003). Thus, activation of Wnt/β-catenin and/or Fgf signalling could account for this novel mid-trunk expression peak.

We confirmed via whole-mount *in situ* hybridisation that *Fgf8* reaches its maximum intensity of expression, as well as anteroposterior length of its expression domain, at E9.5-E10.5, immediately preceding a peak in somite size (Fig. 5D; Fig. S8). At this stage, expression of the RA synthetic enzyme *Aldh1a2* in the somites was maximally separated from the *Fgf8* expression domain at E9.5, suggesting that the known antagonistic relationship between Fgf8 and RA (Diez del Corral et al., 2003; Sirbu and Duester, 2006) scales with the peak and decline in NMP numbers.

### Hox gene expression is upregulated between E8.5-10.5

**Fig. 6.**
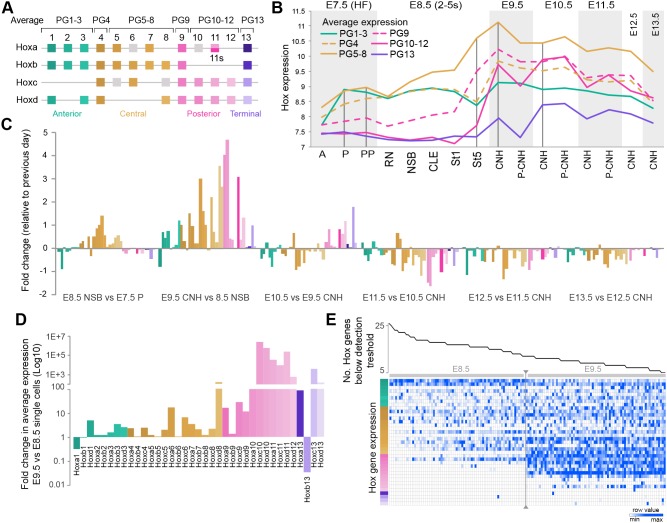
**Hox expression peaks during primitive streak-to-tail bud transition.** (A) Hox gene classification into anterior (green), central (gold), posterior (pink) and terminal groups (purple). (B) Timing of Hox gene expression in different paralogous groups (PGs). Expression of individual genes is shown in Fig. S9A-D. (C) Relative levels of Hox transcripts in NMP-containing regions show highest Hox levels are reached between E8.5 and E9.5 in all but PG13 genes. Colours match those in A. (D) Single CLE cell (Gouti et al., 2017) Hox expression values averaged and represented as E9.5 versus E8.5 fold change. (E) Heatmap of single E8.5-E9.5 CLE cells, organised by the number of Hox genes expressed (columns) versus Hox genes (rows). Coloured bar represents the gene order shown in D.

Several of the genes that were most highly upregulated in NMP- and LPMP-containing regions between E8.5 and E10.5 were members of the Hox gene family, which regulate anteroposterior axial pattern (reviewed by Mallo et al., 2010). Wnt signalling has recently been shown to activate the 3′ (anteriorly expressed) part of the HoxA cluster and facilitate the activation of more 5′, Cdx-dependent, trunk Hox genes (Amin et al., 2016; Neijts et al., 2017). However, no general upregulation has been reported *in vivo* between E8.5 and E10.5. Furthermore, chick Hox clusters are activated in a temporally collinear sequence *in vivo* (Denans et al., 2015) but information on the exact time of activation of these genes in mouse embryos is incomplete. We therefore examined our whole microarray dataset for patterns and timing of Hox activation and further upregulation. Hox genes can be broadly classified into anterior (paralogous groups, PG1-3), central (PG4-8) and posterior (PG9-13) subgroups (reviewed by Young and Deschamps, 2009), with PG13 (here referred to as ‘terminal’) proposed to precipitate the decision to stop axis elongation (Young et al., 2009). After filtering out probes that did not show activation above background (Fig. S9I), genes were grouped according to the above criteria (Fig. 6A).

PG1-3 genes were highly expressed in the E7.5 headfold stage primitive streak. Their expression declined gradually after E9.5 (Fig. 6B; Fig. S9A). The central Hox group (PG4-8) was active at headfold stage, and increased prominently on E8.5. Expression peaked on E9.5 and subsequently declined. PG9-12 expression was low at E7.5, slightly elevated in the E8.5 posterior streak and strongly upregulated at E9.5-E10.5, declining from E11.5 onwards (Fig. 6B; Fig. S9B,C). PG4 and PG9 genes showed profiles intermediate between PG1-3/5-8 and PG4-8/9-12, respectively. PG13 gene expression rose between E8.5 and E10.5, and declined thereafter (Fig. 6B; Fig. S9D). Measuring the extent of change relative to the previous day specifically in the NSB-CNH subpopulation showed that Hox gene expression conformed to the pattern described above (Fig. 6C). Taken together with published data (Forlani et al., 2003; Izpisua-Belmonte et al., 1991; Juan and Ruddle, 2003; Scotti and Kmita, 2012; Soshnikova and Duboule, 2009; Tschopp et al., 2009), this indicates that PG1-4 Hox genes are active before the first time point of E7.5, PG5-8 genes are activated around this time, PG9-12 genes are activated between E8.5 and E9.5, and the terminal Hox genes are activated around E9.5. Despite these generalities, subtle differences between members of a given paralogous group, and cluster-specific profiles were evident (Fig. S9E-H), e.g. the profiles of *Hoxc6*, *Hoxc9* and *Hoxc10* were strikingly similar, possibly reflecting cluster-specific regulation (Neijts et al., 2017, 2016).

As the NSB and CNH samples also contained NotoPs, we confirmed that temporal change in the Hox genes occurs specifically in NMPs by measuring the averaged expression of single E8.5-9.5 CLE cells (Gouti et al., 2017) (Fig. 6D). Furthermore, ordering single cells by the number of Hox genes expressed above the detection threshold showed a remarkably consistent correlation with the position of a given Hox gene in the cluster (Fig. 6E), indicating temporal collinearity in individual CLE cells. Thus, Hox gene expression in NMPs shows a general upregulation between E8.5 and E9.5, with a gradual decline between E10.5 and E13.5 (Fig. 6B,C). Furthermore, temporal collinearity implies that anteroposterior patterning originates in NMPs and LPMPs.

### Ventral NSB cells are a transcriptomically stable, quiescent and static population essential for axis elongation

**Fig. 7.**
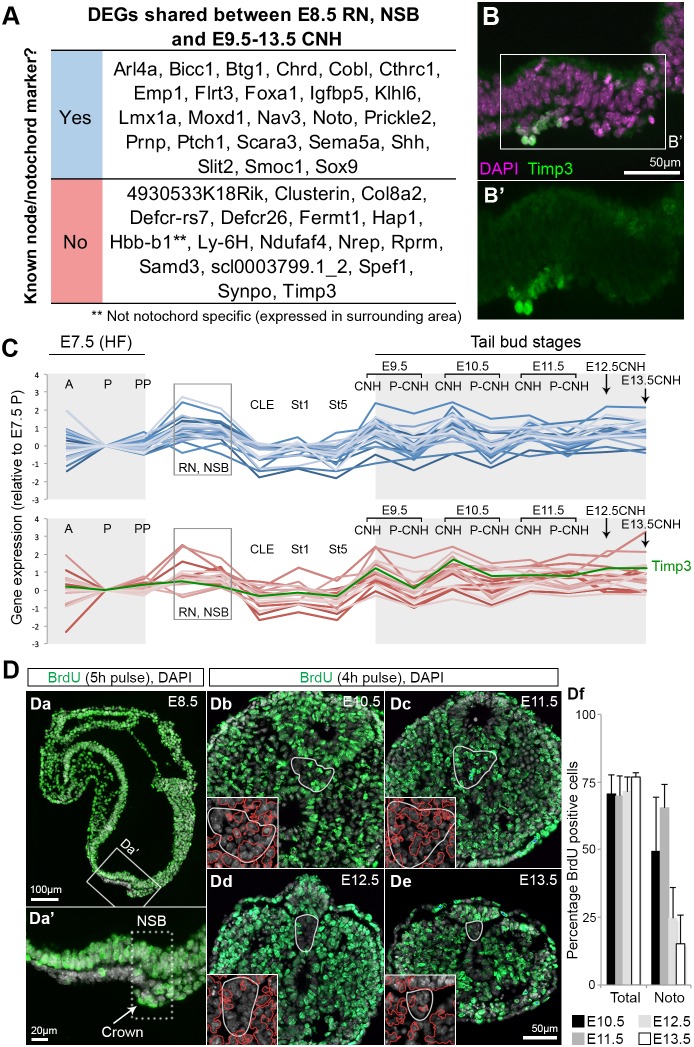
**Quiescence and transcriptional stability of NotoPs.** (A) DEGs shared between E8.5 RN and NSB and E9.5-13.5 CNH samples highlight known, and reveal potential novel, node/notochord markers. Two genes, *Sp6* and *Etsrp71* (*Etv2*), were specifically downregulated. (B) Immunostaining for Timp3 shows elevated expression in the ventral node layer. (C) Genes in A plotted over time. Known markers are in blue; potential novel markers are in red; Timp3, green. (D) Immunohistochemistry detecting incorporated BrdU (green) in E8.5 (2-3 s) embryo and tail sections. White outline indicates the posterior notochord domain. Insets contain DAPI-stained nuclei showing the position of BrdU labelled cells (red). (Df) Quantification of BrdU^+^ cells in tail sections. Data are mean±s.d.; noto, notochord (see also Fig. S10B).

As both NMP and LPMP transcriptomes change with time, we investigated whether the same was true of the NotoP population. At E8.5, genes expressed in the RN-NSB domain included known markers of NotoPs. We therefore searched for genes enriched in RN, NSB and all CNH samples that would constitute NotoP markers. This gene set overlapped extensively with genes enriched in cells expressing the NotoP marker Foxa2 (Tamplin et al., 2011) (Fig. S10A; Table S8). Indeed, about half of the genes fulfilling these criteria (23/41) were previously identified markers of the node and emergent notochord (Fig. 7A). Within the remaining half, we validated one of these novel potential NotoP markers, Timp3, a metalloproteinase inhibitor, via antibody staining (Fig. 7B). Interestingly, the levels of expression of these 41 genes in each NotoP-containing sample were relatively stable over time (Fig. 7C). This raised the possibility that the NotoP population, which at early stages coincides with the organiser of the neuraxis and at all stages contacts NMPs, may stabilise behaviours of adjacent populations throughout axial elongation.

**Fig. 8.**
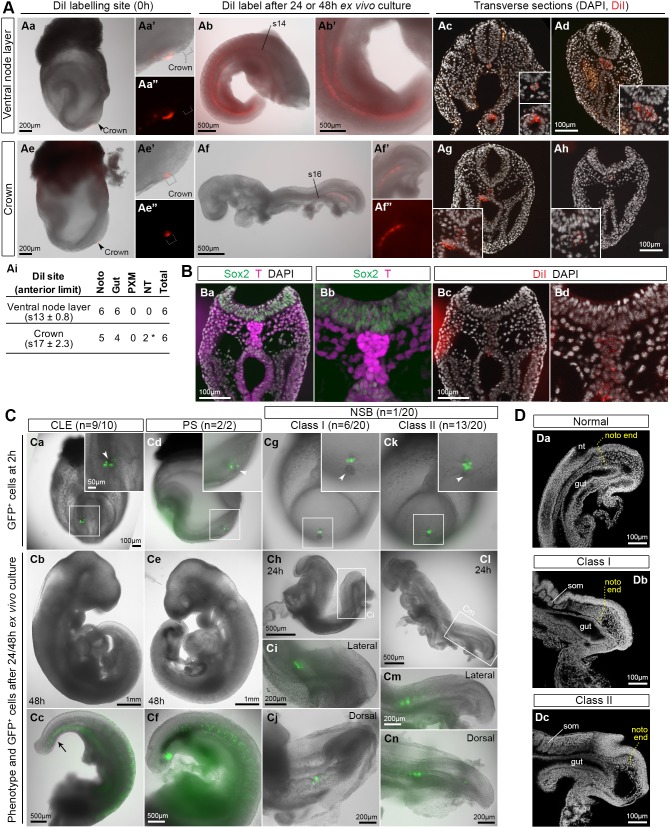
**NotoPs are essential for correct axis elongation.** (A) DiI labelling of the ventral node or crown at E8.5 (2-5 s; Aa,Ae). (Aa′,Ae′) Magnified view of the ventral node region. (Aa″,Ae″) Red channel showing DiI labelling. The same embryos are shown after 48 h (Ab) or 24 h (Af) *ex vivo* culture. Ab′ and Af' show magnified views of Ab and Af respectively. (Af″) Red channel showing DiI labelling. DiI was found in the notochord and dorsal gut (Ac,Ad,Ag,Ah). Insets in Ac, Ad, Ag and Ah show magnified views of DiI labelling in notochord and gut. (Ai) DiI labelling per embryo after culture with average anterior limit (±s.d.). The anterior limit in 4/6 crown-labelled embryos was in the presomitic mesoderm. The anterior limit in each of these embryos is denoted as (total somites/embryo) +1. As the presomitic mesoderm contains ∼7 presomites, the average anterior limit may be posterior to s17. Noto, notochord; NT, neural tube; PXM, paraxial mesoderm. Asterisk indicates 2/6 embryos had a minor contribution in the posterior neural tube. (B) Sox2/T immunostained section of embryo labelled with DiI in the crown after 24 h in culture (n_embryos_=3). (C) Electroporation of GFP-containing plasmid in the CLE (Ca-Cc), primitive streak (Cd-Cf) and NSB (Cg-Cn) of E8.5 (2-5 s) embryos with n, the number of embryos developing normally/total cultured. (Ca-c,Cd-f) Representative CLE- and primitive streak-electroporated embryos, respectively, after 2 or 48 h. (Cg-j,Ck-n) Representative Class I and Class II embryos, respectively, 24 h after NSB electroporation. Arrowheads indicate cell death after electroporation. Black arrow in Cc indicates hindgut label (see Fig. S12). (D) Sagittal confocal sections through CLE-electroporated (Da), and NSB-electroporated class I (Db) and II (Dc) embryos after 24 h, with the notochord (noto) end shown in yellow. nt, neural tube; som, somite.

Consistent with this possibility, previous reports have shown that the ventral node and notochordal plate contain slow-dividing or quiescent cells at E7.5-E8.5 (Bellomo et al., 1996), although they are more proliferative at E9.5 (Ukita et al., 2009). To determine the proliferative characteristics of the crown and later notochordal plate, we analysed cells in S-phase after bromodeoxyuridine (BrdU) labelling. Both the crown at early somite stages and the ≥E10.5 notochordal plate contained a mixture of labelled and unlabelled cells. (Fig. 7Da). Nevertheless the proportion of NotoPs in S-phase relative to other tail bud regions during tail development was low (15-60% versus 70% in the surrounding tissues; Fig. 7Db-Df; Fig. S10B). Although the mouse node has been fate mapped as a whole, the fate of the posterior crown region, which lies just ventral to the NMPs, is unknown. Control DiI label of the ventral node resulted in the expected descendants along the length of the notochord as far posteriorly as the notochord end, as well as the dorsal hindgut (Fig. 8Aa-Ad). Descendants of the crown also populated the dorsal hindgut and notochord, although in a more posterior region than the whole node (Fig. 8Ae-Ai; Fig. S11A). This suggests that crown cells contain NotoPs whose exit from the progenitor region is delayed relative to NotoPs in the rostral node. This is consistent with homotopic grafts of the whole NSB, where descendant cells were found in the notochordal plate and posterior part of the notochord (Cambray and Wilson, 2007). The gut was unlabelled in these grafts, suggesting that DiI additionally labels a neighbouring population of dorsal/posterior endoderm progenitors, whereas the cells in contact with the NSB are exclusively notochord progenitors. Consistently, descendants of the crown remained in contact with NMPs (Fig. 8B, Fig. S11B). Thus, the stable gene expression, low proliferation and retention of NotoP descendants in the posterior end of the notochord suggests that NotoPs are ideally placed to provide stable environmental signals to NMPs.

Highly localised electroporation of mouse epiblast cells is accompanied by a small region of cell death mainly in the adjacent outer layer of cells, i.e. the endoderm or the exposed notochordal plate (Fig. S11C; Huang et al., 2015). We exploited this localised cell ablation to investigate the role of the ventral NSB in axial elongation. Electroporation of control CLE or primitive streak resulted in normal development and widespread distribution of electroporated cell descendants, according to their expected fates: neurectoderm and mesoderm from CLE; and mesoderm from primitive streak (Fig. 8Ca-f). Hindgut labelling was also observed, probably due to plasmid uptake by the endoderm (Fig. S12). In contrast, electroporating the NSB resulted in sparsely labelled embryos (Fig. 8Cg-n; Fig. S12), exhibiting two distinct phenotypes (Table S9): severe (Class I; *n*=6), where axis elongation halted immediately despite apparently viable electroporated putative NMPs dorsal to the crown (Fig. 8Cg-j); and milder (Class II; *n*=13), where embryos failed to turn, the anteroposterior axis was moderately foreshortened and kinked, and the notochordal plate was wider, ending further anteriorly than in control embryos (Fig. 8Ck-n,D). Electroporated cell descendants populated neurectoderm and occasionally mesoderm (Fig. 8Cj,n; Fig. S12), and were rarely found in the progenitor region. Class II phenotypes were recapitulated in embryos where a small area including the ventral NSB layer had been manually removed, but not in controls where an equivalent area of endoderm under the mid-primitive streak was removed (Fig. S13). Thus, the E8.5 crown region of the ventral node is essential for normal axial elongation. Taken together, these data suggest that the E8.5 crown, and its descendant the posterior notochordal plate, provide a stable environment important for axis elongation, and thus may constitute the equivalent of a ‘niche’ for NMPs.

## DISCUSSION

**Fig. 9.**
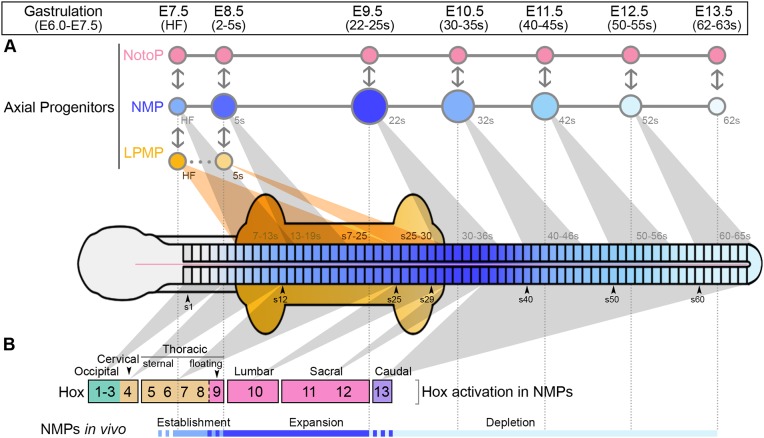
**Summary.** Model showing the progression of NMP (blue), LPMP (yellow) and NotoP (pink) populations during axis elongation, the activation of Hox genes and regulatory changes in NMPs over time. (A) Changes in size of the respective populations are indicated by diameter of the circles at each stage; colour changes represent transcriptomic shifts. Contribution of NMPs and LPMPs to specific axial levels is indicated by grey and orange shading, respectively (e.g. an NMP at the 22 s stage will contribute to axial structures at ∼30-36 s) (data from Cambray and Wilson, 2007; Castillo et al., 2016; Tam, 1986; Wymeersch et al., 2016). Double-headed arrows indicate interactions between populations. (B) Activation of Hox genes in NMPs and the vertebrae they pattern (based on Burke et al., 1995; Kuratani, 2009). Colours indicate the paralogous group classifications shown in Fig. 6.

Comprehensive spatiotemporal analysis of progenitor populations provides novel insights into the progressive production of tissues along the anteroposterior and mediolateral axes of the mouse embryo. We identified characteristic transcriptomes of three known progenitor populations, the NMPs, LPMPs and NotoPs, and discovered major transcriptional shifts in the NMP and LPMP populations during axis elongation. In contrast, the adjacent NotoP population has a largely unchanging trancriptome over this time period, and we propose that they act as a ‘niche’ for NMPs (Fig. 9).

### NMPs, LPMPs and NotoPs are defined by distinct transcriptomes

The three spatial domains identified by transcriptome analysis at E8.5 correspond well with the three axial progenitor cell types previously identified by fate-mapping studies (Cambray and Wilson, 2007; Wilson and Beddington, 1996; Wymeersch et al., 2016). Cells of the CLE have an almost identical transcriptomic profile to their immediate mesoderm-committed descendants in the streak midline. Interestingly, comparison of the transcriptome of NMPs in the chick CLE (stem zone) with their immediate neural-committed descendants in the pre-neural tube also reveals that the majority of changes occur after neural commitment, during differentiation of the pre-neural tube to the neural tube (Olivera-Martinez et al., 2014). As gene expression and function are extensively conserved between mouse and chick, NMP commitment to either neurectoderm or mesoderm may involve only minor transcriptional differences.

Consistent with this idea, the only ≥1.5-fold DEGs between NMPs and mesoderm-committed primitive streak cells are *Tbx6* and *Dll1*, which are upregulated in the anterior primitive streak. Expression of these genes is instrumental in paraxial mesoderm differentiation. Tbx6 enforces paraxial mesoderm differentiation of presumptive NMPs: null mutations in this gene lead to the neural differentiation of already ingressed prospective paraxial mesoderm (Chapman and Papaioannou, 1998; Takemoto et al., 2011). A pivotal position of Tbx6 in mesoderm commitment of NMPs is also suggested by the reciprocal expression of Tbx6 and the NMP marker Sox2 *in vitro* (Gouti et al., 2017). *Dll1*, a Notch ligand involved in somite differentiation, is a known target of Tbx6 (Hrabe de Angelis et al., 1997; White and Chapman, 2005). As Tbx6 also functions downstream of Notch signalling (White et al., 2005), these data suggest Notch signalling promotes paraxial mesoderm commitment of NMPs, while Dll1 upregulation may further reinforce it. Furthermore, several genes that are <1.5-fold upregulated in the primitive streak are also associated with Notch signalling (Fig. S3B), supporting a role for this pathway in mesoderm commitment of NMPs.

### Temporal change in LPMPs and NMPs

Our analysis of E7.5 and E8.5 LPMPs highlighted profound temporal changes. Although the posterior primitive streak analysed in our study at headfold stage has not been fate mapped, the posterior streak at the slightly earlier bud stage gives rise to interlimb LPM and, at low frequency, to cells in the E8.5 posterior primitive streak (Kinder et al., 1999; Smith et al., 1994). Therefore there is probably an overlap between LPMPs at E7.5 and E8.5. However, it is unclear whether the divergent transcriptome between the two regions reflects a single population undergoing maturation (i.e. progressive temporal change in progenitors) or two largely separate cell populations.

In contrast, clonal and population fate mapping shows that late-stage NMPs are largely derived from earlier NMPs (Cambray and Wilson, 2007; Tzouanacou et al., 2009). Therefore, the temporal change in NMP-containing regions implies the maturation of individual NMPs throughout axial elongation. This indicates that the changing intrinsic properties of NMPs (indicated by collinear Hox expression in NMPs) may result in regionalisation of their differentiated derivatives along the axis. Interestingly, Hox expression promotes limb bud outgrowth (Davis et al., 1995; Kmita et al., 2005), suggesting that the general upregulation of Hox genes between E8.5 and E9.5 may be related to the peak in NMP numbers at E9.5 and subsequent expansion of somite size.

### A stable niche for axial progenitors

Our observation that ablation of the posterior crown of the node impairs axis elongation recalls experiments carried out in chick (Charrier et al., 1999), which show that at early somitogenesis stages, ablation of both layers of an area approximately equivalent to the mouse NSB also leads to termination of axial elongation. Together, these experiments point to an essential, evolutionarily conserved role of the crown cells in axial elongation. Interestingly, transplantation of cells from the predominantly transient CLE population to the NSB leads to their retention in the CNH (Wymeersch et al., 2016), the only region that is serially transplantable between generations of cultured embryos and can thus be considered resident (Cambray and Wilson, 2002). Therefore, the crown cells may provide a ‘niche’ that anchors NMPs in the progenitor region. This does not rule out other roles such as in left/right patterning, suggested by the failure of crown-ablated embryos to turn. Although it remains to be determined whether the later notochordal plate organises axial elongation after E8.5 in the same way as the crown cells, the aberrant tail phenotype of embryos where NotoPs are missing (Abdelkhalek et al., 2004) suggests that they are required for later axial elongation.

This ‘niche’ for NMPs at the node/streak border is the direct descendant of the Spemann organiser equivalent in the earlier embryo: the node (Beddington, 1994; Kinder et al., 1999). The *Xenopus* Spemann organizer, like the mouse node, consists almost exclusively of notochord progenitors (Beddington, 1994; Smith and Slack, 1983), suggesting that it is the equivalent of the NotoP population in mouse. Our finding that NMPs show dynamic gene expression changes, including in Hox expression, while the underlying notochordal plate does not, suggests a model in which anteroposterior axial patterning, via the sequential expression of progressively more posterior genes, is intrinsic to NMPs rather than the organiser. These data are interesting in the context of experiments showing that the *Xenopus* Spemann organiser induces anterior neural character in overlying ectoderm but not detailed anteroposterior pattern (Jansen et al., 2007). Instead, pre-ingression non-organizer mesoderm (the topological equivalent of NMPs and LPMPs), shows intrinsic anteroposterior patterning (reviewed by Durston et al., 2010).

Transfer to a ‘young’ NMP environment can reset ‘old’ NMP expression (McGrew et al., 2008). How can this observation be reconciled with an intrinsic timing mechanism in NMPs? Community effects may operate, whereby the number of similar surrounding cells determines whether introduced cells self-differentiate or integrate with their surroundings (Gurdon, 1988; Huang et al., 2012, 2015; McGrew et al., 2008; Trainor and Krumlauf, 2000). This is supported by the observation that, in this population, lack of Cdx2 can be overcome by neighbouring cells (Bialecka et al., 2010), and in zebrafish, axial progenitors (which presumably include NMPs) create a Wnt-dependent environment whereby Brachyury mutation can be tolerated (Martin and Kimelman, 2010). In this scenario, the intrinsic timing of NMPs would respond to changes in local extracellular signalling.

In conclusion, we hypothesise that the vertebrate NotoP population, besides producing notochord, serves as a stable point for organisation of NMPs throughout axial elongation, whereas the NMPs, via Wnt-dependent community effects, undergo maturation. This leads to an expansion of progenitor numbers, an increase in their expression of Wnt, Fgf and Notch signalling pathway components, as well as a quantitative increase in Hox genes of all categories from E8.5-E9.5; this sets up the progenitor pool for sacral/caudal somite production and the activation of terminal Hox genes.

## MATERIALS AND METHODS

### Mouse strains, staging and husbandry

Wild-type, outbred MF1 mice were used for microarray samples. *sGFP* conditional reporter transgenic (Gilchrist et al., 2003) or MF1 mice were used for electroporation. All mice were maintained on a 12 h light/12 h dark cycle. For timed matings, noon on the day of finding a vaginal plug was designated as E0.5. Staging of early mouse embryos was carried out according to Downs and Davies (1993). All animal experiments were performed under the UK Home Office project license PPL60/4435, approved by the Animal Welfare and Ethical Review Panel of the University of Edinburgh and within the conditions of the Animals (Scientific Procedures) Act 1986.

### Microdissection and sample preparation

Microdissection of embryonic regions was performed as described previously (Cambray and Wilson, 2007; Wymeersch et al., 2016). Embryonic regions of a single type were pooled to constitute one sample with at least two replicate samples per embryonic region (Fig. S1A). Specifically, we collected three regions in E7.5 embryos: an anterior neural-fated region (A); and a posterior region comprising the rest of the embryo, including the primitive streak (P) and the posteriormost primitive streak (PP). At E8.5, we analysed the rostral node (RN), node-streak border (NSB), the rostral 1/5 of the primitive streak (St1), the rostral 3/5 of the caudal lateral epiblast (CLE) and the posterior 1/5 of the primitive streak (St5). The CLE samples correspond to the L1-3 region in Wymeersch et al. (2016), where the underlying presomitic mesoderm and endoderm were dissected away from the ectoderm. Regions at subsequent stages up to E11.5 included the chordoneural hinge (CNH), and the region immediately posterior to the CNH (P-CNH). At E12.5-E13.5, owing to the small size of the P-CNH region, only the CNH region was collected. Whole regions were isolated rather than germ layer-dissected tissue to ensure as fast a workflow as possible. This meant that along with the target cell types, several expected minor populations were present. For example, endoderm was present in all <E8.5 samples, except CLE, whereas surface ectoderm was expected to be present in St5 and E13.5 CNH. However, in no case was a non-target tissue uniquely associated with a single cell type of interest, and therefore the data could be used to draw conclusions about expression profiles in the target cell types.

### Microarray analysis

RNA was isolated using the RNeasy Micro Kit (Qiagen) and labelled and amplified using the Illumina TotalPrep RNA Amplification Kit (Life Technologies). The sample concentrations and quality were determined using a 2100 bioanalyzer (Agilent). Samples were loaded on six MouseWG-6 v2.0 Expression BeadChip arrays (Illumina). Data normalisation was performed using the lumi package in the R statistical environment (Du et al., 2008). Pre-processing steps consisted of a background adjustment, followed by Variance-Stabilizing Transformation (VST) and Robust Spline Normalization (RSN). A final quality control step was carried out to detect outliers, and probes that were not expressed in any samples were filtered (23,569 out of 45,281 probes; detection *P*-value<0.01). ComBat analysis (Chen et al., 2011) was used to correct for any batch effects. DEGs were identified using the limma package (parameters: BH with fold-change ≥1.5 and FDR ≤0.05; Smyth, 2004). Marker expression in individual microdissected pieces has been shown previously (Cambray and Wilson, 2007; Wymeersch et al., 2016) and is validated by qRT-PCR and *in situ* hybridisation here (Fig. S1; Fig. S2). Hierarchical clustering was performed using the Morpheus visualisation tool (software.broadinstitute.org/morpheus). To assign genes of interest to a specific signalling pathway or cellular process, we used the Kyoto Encyclopedia of Genes and Genomes (Kanehisa et al., 2016) and the STRING database (Franceschini et al., 2013).

### *In situ* hybridisation

Whole-mount *in situ* hybridisation was performed as described previously (Wilkinson, 1998) except that proteinase K treatment was empirically adjusted according to embryo size and stage (time between 5-20 min). Riboprobes were designed against *Bhmt2* (NM_022884.1, nt1189-1917), *Ccno* (NM_001081062.1, nt79-1048), *Rspo3* (NM028351.3, nt628-1740) and *Sall4* (NM201396.2, nt515-984) mRNA sequences. Other riboprobes used included: *Aldh1a2* (Zhao et al., 1996), *Dusp6* (Dickinson et al., 2002), *Fgf8* (Mahmood et al., 1995), *Fgf17* (Maruoka et al., 1998), *Mnx-1* (Szumska et al., 2008), *Shh* (Echelard et al., 1993) and *Wnt3a* (Takada et al., 1994). Measurements of somite length and *Fgf8* and *Aldh1a2* expression domain length were performed on whole-mount images using Volocity software (Perkin Elmer).

### Quantitative RT-PCR

For microarray validation, ∼10-15 independently dissected regions of the primitive streak were pooled to make up one sample. Total RNA was isolated using a RNeasy microkit (Qiagen) and cDNA synthesis performed using SuperScript III (Life Technologies). qRT-PCR was performed using Light Cycler 480 SYBR Green I Master Mix (Roche). Expression values were normalized to the expression of the TATA-box binding protein (TBP). Primer sequences can be found in Table S10.

### Immunohistochemistry

Embryo cryosectioning, staining and immunofluorescence was performed as described previously (Huang et al., 2012). Primary antibodies (supplier, catalogue number and working concentration) were: anti-Timp3-loop1 (Abcam; ab39184; 5 µg/ml), anti-Sox2 (Abcam; ab92494; 1:200), anti-T (R&D; AF2085; 1 mg/ml) and anti-GFP (Abcam; ab13970; 10 mg/ml). For S-phase analysis, E8.5 (2-5 s) embryos were cultured *ex vivo* in rat serum-containing medium at 37°C for 5 h, containing 31 µg/ml BrdU (BD Biosciences) (Bellomo et al., 1996). Similarly, tail buds (including the PSM and the last two formed somite pairs) from E10.5-E13.5 embryos were cultured for 4 h, but in N2B27 culture medium (Invitrogen). Samples were fixed overnight in 4% paraformaldehyde in PBS at 4°C and cryosectioned. Antigen retrieval was performed with 10 mM sodium citrate (pH 6.0) for 10 min (Tang et al., 2007) and sections were stained with a BrdU Labelling and Detection Kit I (Roche). Cells were counted using Photoshop (Adobe) and ImageJ software (NIH).

### Embryo manipulations

Fluorescent cell tracking was performed with CellTracker CM-DiI (Thermo Fisher Scientific) as described previously (Wilson and Beddington, 1996). Electroporation of pCAG-GFP or pCAG-Cre:GFP plasmids was performed on E8.5 (2-5 s) wild-type or *sGFP* embryos, respectively, using an optimized electroporation method to target small numbers of cells (Huang et al., 2015). Cell death analysis was performed with DRAQ7 dye (Abcam) according to manufacturer's instructions. *Ex vivo* whole-mount embryo culture was performed as described previously (Copp and Cockroft, 1990). After 24/48 h, cultured embryos were dissected, imaged and scored on phenotype and GFP contribution. Scoring criteria for Class I embryos were: failure to elongate and turn; head truncation or malformation; kinked neural tube; and small somites. Class II embryo criteria were: failure to turn; moderate elongation with somite formation; kinked neural tube; open posterior neural plate; and/or small tail bud. Additional electroporation details can be found in Table S9. To remove the ventral cell layer at the NSB or St3, a sharp glass needle was inserted from posterior to the region of interest and pulled ventrally to separate ventral and dorsal cells. The free cell layer was trimmed to remove the crown or St3 endoderm, after which DiI was poured on the site, labelling all exposed cells (Fig. S13).

### Image analysis

Whole-mount embryo images were taken on a Nikon AZ100 (Nikon) or Leica M165 FC microscope (Leica). A wide-field Olympus BX61 or Zeiss Observer microscope with fluorescence optics were used to capture images of immunostained cryosections. Confocal imaging was acquired on a Leica TCS SP8 platform (Leica). Image processing was carried out using Adobe Photoshop (Adobe Systems) and ImageJ software (Schneider et al., 2012).

## Supplementary Material

Supplementary information
