# Supplementary material for: Transcriptionally dynamic progenitor populations organised around a stable niche drive axial patterning
Source: Development. 2019 Jan 2;146(1):dev168161. doi: 10.1242/dev.168161 (PMC6340148; doi:10.1242/dev.168161)
Supplement: Supplementary information [file develop-146-168161-s1.pdf]

**A**

| Age | Stage | Region | NotoP | NMP | LPMP | Nascent presomitic mesoderm | Nascent lateral mesoderm | Anterior neurectoderm | Notochord | Endoderm | Ventral neurectoderm | Surface ectoderm | Presomitic mesoderm | Somite | Blood vessel | replicates | no. regions per replicate | | | Replicate Naming | | |
|---|---|---|---|---|---|---|---|---|---|---|---|---|---|---|---|---|---|---|---|---|---|---|
| | | | **Major components** | | | | | | | **Minor components** | | | | | | | | | | | | |
| E7.5 | HF | A | | | | | | ✓ | ✓ | ✓ | | | | | | 3 | 9 | 13 | 25 | 7A1 | 7A2 | 7A3 |
| | | P | ✓ | (✓) | ✓ | ✓ | ✓ | | | ✓ | | | | | | 2 | 9 | 13 | - | 7P1 | 7P2 | |
| | | PP | | | ✓ | | | | | ✓ | | | | | | 2 | 12 | 15 | - | 7PP10 | 7PP15 | |
| E8.5 | 2-5s | RN | ✓ | | | | | | | ✓ | ✓ | | | ✓ | | 2 | 45 | 45 | - | RN-1 | RN-2 | |
| | | NSB | ✓ | ✓ | | | | | | ✓ | | | | | | 3 | 45 | 45 | 57 | Bord-1 | Bord-2 | 8B3 |
| | | CLE | | ✓ | | | | | | | | | ✓ | | | 3 | 12 | 43 | 36 | 8L1 | 8L2 | 8L3 |
| | | St1 | | | | ✓ | | | | ✓ | | | | | | 2 | 45 | 45 | - | St1-1 | St1-2 | |
| | | St5 | | | ✓ | | ✓ | | | ✓ | | ✓ | | | | 2 | 45 | 45 | - | St5-1 | St5-2 | |
| 9.5 | 22-25s | CNH | ✓ | ✓ | | | | | | | | | | | | 2 | 6 | 10 | - | 9M1 | 9M2 | |
| | | P-CNH | | | | ✓ | | | | | | | | | ✓ | 2 | 6 | 11 | - | 9P1 | 9P2 | |
| E10.5 | 32-35s | CNH | ✓ | ✓ | | | | | | | | | | | | 2 | 17 | 17 | - | MCNH-1 | MCNH-2 | |
| | | P-CNH | | | | ✓ | | | | | | | | | ✓ | 2 | 17 | 17 | - | PCNH-1 | PCNH-2 | |
| 11.5 | 40-45s | CNH | ✓ | ✓ | | | | | | | | | | | | 3 | 38 | 29 | 52 | 11M1 | 11M2 | 11M3 |
| | | P-CNH | | | | ✓ | | | | | | | | | ✓ | 2 | 33 | 65 | - | 11P1 | 11P2 | |
| 12.5 | 50-55s | CNH | ✓ | ✓ | | | | | | | | | | | | 2 | 15 | 33 | - | 12M1 | 12M2 | |
| 13.5 | 60-63s | CNH | (✓) | (✓) | | | | | | | | ✓ | | | ✓ | 2 | 48 | 74 | - | 13M1 | 13M2 | |

Microarray 1, Microarray 2 — Batch 1
Microarray 3, Microarray 4 — Batch 2
Microarray 5, Microarray 6 — Batch 3

**B**

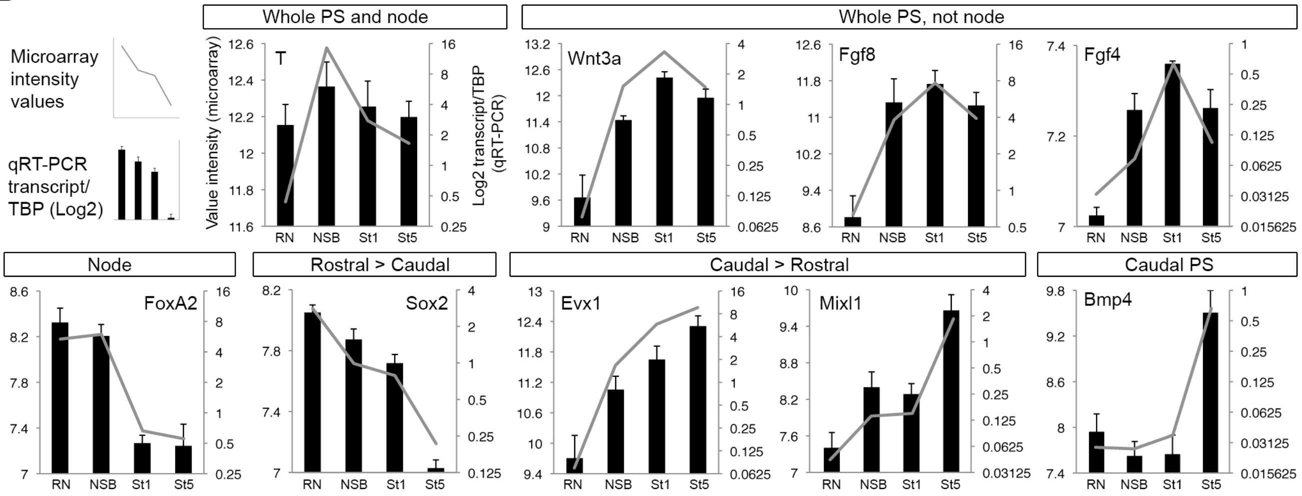


**Figure S1. Microarray sample specifics and qRT-PCR validation.**
(A) Table of dissected samples, showing major and minor cell components included in the samples. A tick indicates presence, brackets indicate uncertainty about the proportion of progenitors in the sample. Replicates were named as shown and run on six different arrays in three batches. Colour of the sample corresponds to the microarray and batch. (B) Microarray validation on independently-dissected samples consisting of ~10-15 pooled regions of the primitive streak (PS). qRT-PCR values were normalised to TBP, the mean ± s.e.m. plotted as log2 values (black bars) and compared to the normalised intensity values of the microarray (grey line).

Development • Supplementary information

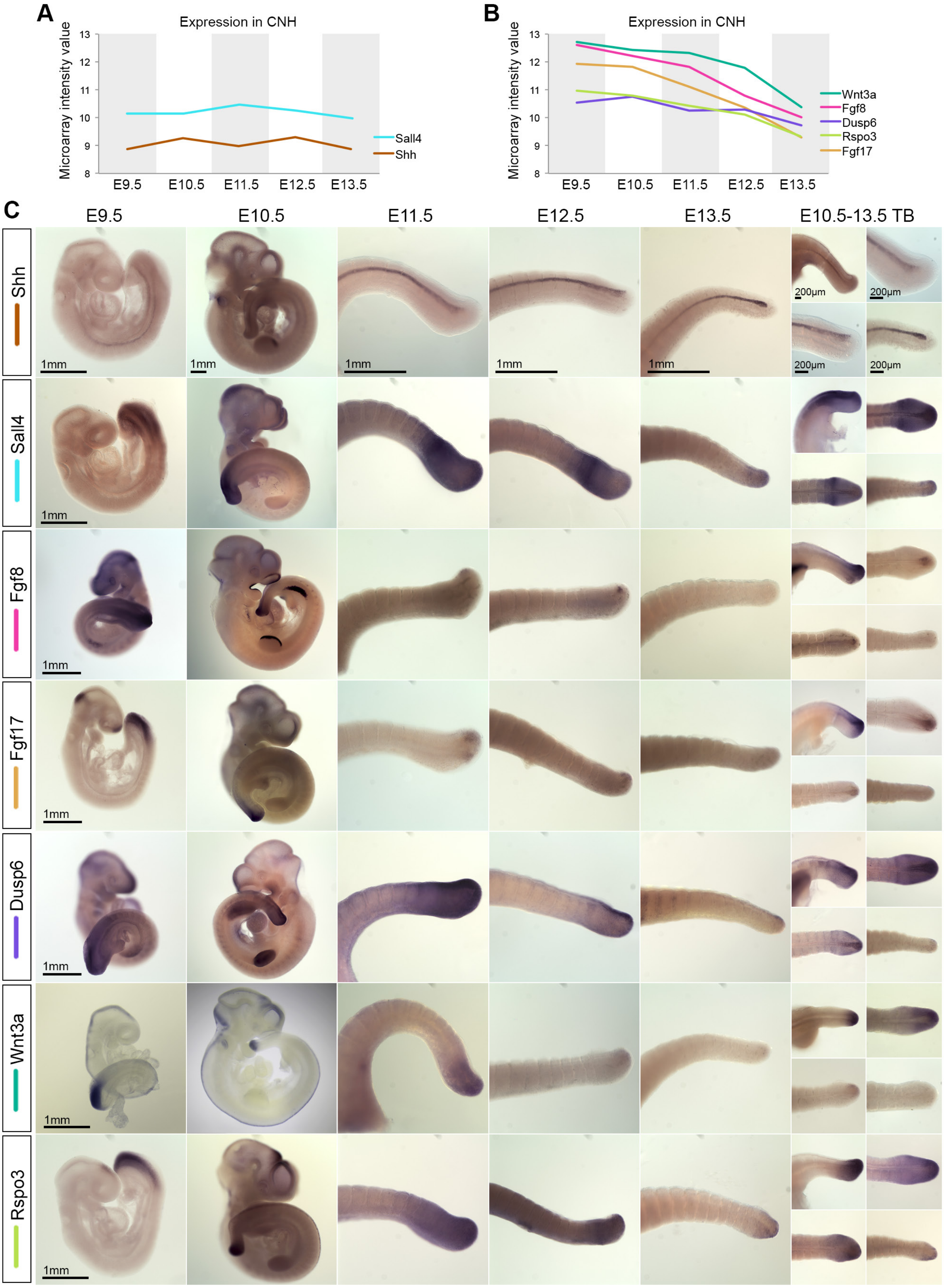


**Figure S2. Validation of microarray by in situ hybridisation.**
(A-C) Microarray expression values for selected marker genes in the CNH. (A) *Sall4* and *Shh* are stable over time, whereas *Wnt3a, Fgf8, Dusp6, Rspo3* and *Fgf17* decline during tail elongation (B). Microarray intensity values correlate well to *in situ* hybridisation data of the caudal progenitor area between E9.5 and E13.5 (C). CNH, chordoneural hinge; TB, tail bud.

Development • Supplementary information

**A**

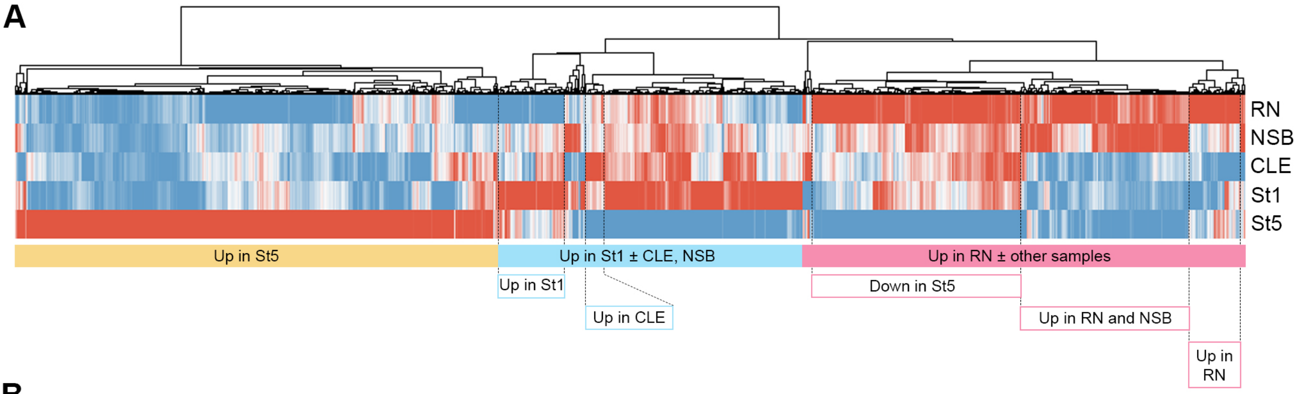


**B**

| | GO-term | Description | p-value | KEGG ID | Description | p-value | Genes |
|---|---|---|---|---|---|---|---|
| **Up in RN ± other samples** | GO.0030182 | neuron differentiation | 9.79E-13 | 5217 | Basal cell carcinoma | 0.0112 | Fzd2, Fzd6, Fzd7, Gli2, Ptch1, Shh, Stk36, Tcf7l2, Wnt8a |
| | GO.0044782 | cilium organization | 4.13E-14 | | | | |
| | GO.0098609 | cell-cell adhesion | 0.0195 | 100 | Steroid biosynthesis | 0.0302 | Dhcr24, Dhcr7, Lss, Sc4mol, Sqle |
| | GO.0030336 | negative regulation of cell migration | 1.65E-05 | 4512 | ECM-receptor interaction | 0.033 | Agrn, Cd47, Col2a1, Col4a2, Dag1, Itga3, Itga6, Reln, Thbs3, Vtn |
| | GO.0030178 | negative regulation of Wnt signaling pathway | 8.40E-06 | | | | |
| | GO.0007368 | determination of left/right symmetry | 1.16E-06 | | | | |
| | GO.0006694 | steroid biosynthetic process | 0.000554 | | | | |
| | GO.0016125 | sterol metabolic process | 0.00179 | | | | |
| | GO.0007219 | Notch signaling pathway | 0.00344 | | | | |
| | GO.0007492 | endoderm development | 0.000428 | | | | |
| | GO.0007224 | smoothened signaling pathway | 0.000674 | | | | |
| | GO.0008203 | cholesterol metabolic process | 0.00402 | | | | |
| | GO.0021517 | ventral spinal cord development | 0.000397 | | | | |
| | GO.0030903 | notochord development | 0.000633 | | | | |
| | GO.0035567 | non-canonical Wnt signaling pathway | 0.012 | | | | |
| | GO.0060071 | Wnt signaling pathway, planar cell polarity pathway | 0.00793 | | | | |
| | GO.1903053 | regulation of extracellular matrix organization | 0.00793 | | | | |
| | GO.0033504 | floor plate development | 0.0011 | | | | |
| | GO.0010873 | positive regulation of cholesterol esterification | 0.0224 | | | | |
| **Up in St1 ± CLE, NSB** | GO.0009893 | positive regulation of metabolic process | 1.30E-06 | 4310 | Wnt signaling pathway | 0.00398 | Ctbp2, Dkk4, Dvl1, Frat2, Fzd10, Fzd5, Gpc4, Sox17, Tcf7, Trp53, Wnt10a, |
| | GO.0016055 | Wnt signaling pathway | 1.76E-05 | | | | |
| | GO.0001843 | neural tube closure | 0.000412 | 4330 | Notch signaling pathway | 0.0217 | Aph1a, Ctbp2, Dll1, Dvl1, Kat2a, Ncstn |
| | GO.0036342 | post-anal tail morphogenesis | 0.00463 | 4014 | Ras signaling pathway | 0.0301 | Angpt2, Epha2, Ets2, Fgf15, Fgf17, Fgf8, Hras1, Plcg2, Rapgef5, Rasa1, Rasa3, Rgl2, Vegfc |
| | GO.0007155 | cell adhesion | 0.0181 | | | | |
| | GO.0016477 | cell migration | 0.0198 | | | | |
| | GO.0030155 | regulation of cell adhesion | 0.0259 | 4015 | Rap1 signaling pathway | 0.047 | Angpt2, Epha2, Fgf15, Fgf17, Fgf8, Gnai2, Hras1, Mapk11, Mapk12, Rapgef5, Src, Vegfc |
| | GO.0001839 | neural plate morphogenesis | 0.0261 | | | | |
| | GO.0072331 | signal transduction by p53 class mediator | 0.0316 | | | | |
| | GO.0046328 | regulation of JNK cascade | 0.0372 | | | | |
| **Up in St5** | GO.0001944 | vasculature development | 4.87E-24 | 4390 | Hippo signaling pathway | 7.94E-07 | Actb, Axin2, Bmp4, Bmp7, Bmpr2, Csnk1d, Frmd6, Id1, Id2, Lef1, Nkd1, Ppp1ca, Ppp2r1a, Snai2, Tgfb1, Tgfb2, Tgfbr2, Wnt4, Wnt5a, Wnt5b, Wnt6, Wtip |
| | GO.0042981 | regulation of apoptotic process | 2.41E-13 | | | | |
| | GO.0043066 | negative regulation of apoptotic process | 3.84E-13 | | | | |
| | GO.0007155 | cell adhesion | 3.27E-09 | | | | |
| | GO.0060173 | limb development | 1.23E-06 | 4350 | TGF-beta signaling pathway | 2.82E-05 | Bmp4, Bmp7, Bmpr2, Id1, Id2, Id3, Id4, Ltbp1, Pitx2, Ppp2r1a, Smad6, Tgfb1, Tgfb2, Tgfbr2 |
| | GO.0043408 | regulation of MAPK cascade | 0.000307 | | | | |
| | GO.0090090 | negative regulation of canonical Wnt signaling pathway | 0.00115 | | | | |
| | GO.0038084 | vascular endothelial growth factor signaling pathway | 0.00155 | 4015 | Rap1 signaling pathway | 0.000111 | Actb, Adora2b, Akt3, Arap3, Ctnnd1, Efna1, Efna4, F2r, Fgf10, Flt1, Id1, Kit, Ngfr, Pdgfra, Pfn1, Prkd2, Prkd3, Ralb, Rasgrp3, Tek, Tiam1, Vegfa |
| | GO.0045746 | negative regulation of Notch signaling pathway | 0.00198 | | | | |
| | GO.0003401 | axis elongation | 0.00455 | | | | |
| | GO.0030509 | BMP signaling pathway | 0.0365 | | | | |
| | | | | 4151 | PI3K-Akt signaling pathway | 0.000214 | Akt3, Ccne2, Cdkn1a, Col24a1, Efna1, Efna4, F2r, Fgf10, Flt1, Fn1, Ghr, Gnb4, Gng12, Gng2, Hsp90ab1, Itga11, Itga5, Itga9, Itgb5, Kit, Lamc1, Ngfr, Pdgfra, Ppp2r1a, Ppp2r5c, Sgk3, Tek, Vegfa, Vwf |
| | | | | 4014 | Ras signaling pathway | 0.00392 | Akt3, Efna1, Efna4, Ets1, Fgf10, Flt1, Gab1, Gnb4, Gng12, Gng2, Kit, Ngfr, Pdgfra, Ptpn11, Ralb, Rasgrp3, Tek, Tiam1, Vegfa |
| | | | | 4115 | p53 signaling pathway | 0.0217 | Bid, Ccne2, Cdk1, Cdkn1a, Gadd45g, Igfbp3, Sesn1, Shisa5 |
| | | | | 4512 | ECM-receptor interaction | 0.0292 | Col24a1, Fn1, Hspg2, Itga11, Itga5, Itga9, Itgb5, Lamc1, Vwf |
| | | | | 4010 | MAPK signaling pathway | 0.0449 | Akt3, Dusp1, Dusp4, Dusp5, Fgf10, Flnc, Gadd45g, Gng12, Map4k4, Nfatc1, Pdgfra, Ppp3ca, Rasgrp3, Rps6ka1, Tgfb1, Tgfb2, Tgfbr2 |

**Figure S3. Gene expression patterns in the E8.5 primitive streak.**
(A) Unsupervised hierarchical clustering of all genes that showed ≥1.5 fold change across E8.5 samples. Three major clusters were identified: up in RN ± other samples (pink), up in St1 ± CLE, NSB (blue) and up in St5 (yellow). In the first two clusters, we further distinguish several subpatterns (see Table S1). (B) Overview of enriched GO-terms and KEGG pathways from patterns in A.

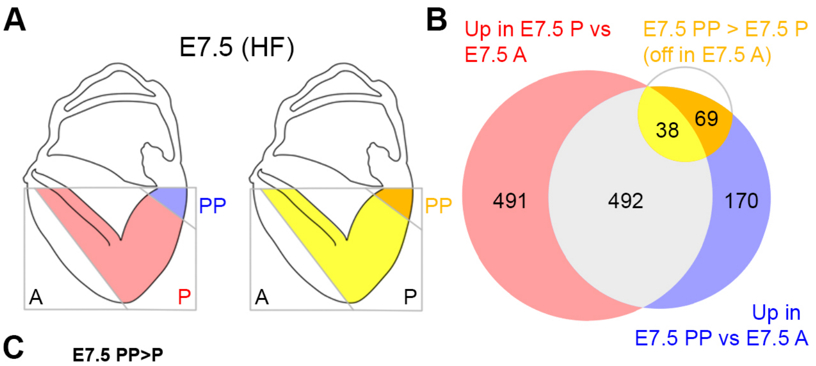


**C**

| E7.5 PP>P | |
|---|---|
| **Included in E7.5 P (≥BMP4 levels)** | Apoa1, Apoa2, Apob, Apom, Bmp4, Clic6, Cotl1, Ctsh, Dab2, Dlk1, Dusp4, Efna1, Ets1, Gal, Ifitm1, Igfbp3, Lin28, Lxn, Mixl1, Mogat2, Mrg1, Msx1, Nefm, Nfkbiz, Nkx1-2, Nme7, Pabpc1, Pmp22, Ppic, Rap2c, Rn18s, Sct, Slc13a4, Supt16h, Tcf7, Tmpo, Ttr, Vps35 |
| **Excluded from E7.5 P (<BMP4 levels)** | 2700060E02Rik, 2810026P18Rik, 2810043G13Rik, 6330419J24Rik, Abca3, Acbd3, Apoe, Atf4, Brd7, C1qbp, Car4, Cdc16, Comt, Crlf1, Ctsb, Cubn, Dkk3, Dnajb11, Dtnbp1, E430033B07Rik, Eef1e1, EG665369, EG666756, Egr1, Etsrp71, Fbxl14, Ghr, Gng12, Hadhb, Hist1h2ah, Hoxd8, Hoxd9, Hsp90aa1, Klhl6, Lamp2, LOC100039786, LOC100042514, LOC100046298, LOC100048436, LOC212399, LOC245892, LOC380665, LOC666053, Mrps6, Myo5b, Ndufs4, Npm3, Pcgf5, Plac1, Psma4, Rbp4, Rdh11, Reep6, Rhox5, S100a11, Sfrs11, Skil, Slc11a1, Slpi, Soat1, Spcs1, Spin2, Spink3, Tbx4, Tcof1, Tdh, Tubb6, Uap1l1, Uchl3 |

**Figure S4. Comparison of LPMPs at E7.5 and E8.5.**
(A) Schematic overview of dissected and compared regions at E7.5 headfold stage (HF). Since E7.5 P overlaps with PP gene expression, samples were compared as follows: E7.5 P versus A (red), E7.5 PP versus A (blue) and E7.5 PP versus P (shades of yellow). Since PP is included in the P sample and thus no gene will be ON in PP and OFF in P, we used the values of a known marker of the PP region, *Bmp4* (Lawson and Wilson, 2016), as the minimum cut-off for transcripts enriched in PP versus P. (B) Overlap of E7.5 sample comparisons with genes specific for E7.5 PP shown in (C) ‘Included’ genes, i.e. ≥BMP4 values, are most likely upregulated in PP, while some of the remainder may also be upregulated there, although less strongly.

**Lawson, K. A. and Wilson, V.** (2016). A Revised Staging of Mouse Development Before Organogenesis. In Kaufman's Atlas of Mouse Development Supplement (ed. B. R., B. J., D. D. & M.-K. G.), pp. 51–64: Elsevier.

Development • Supplementary information

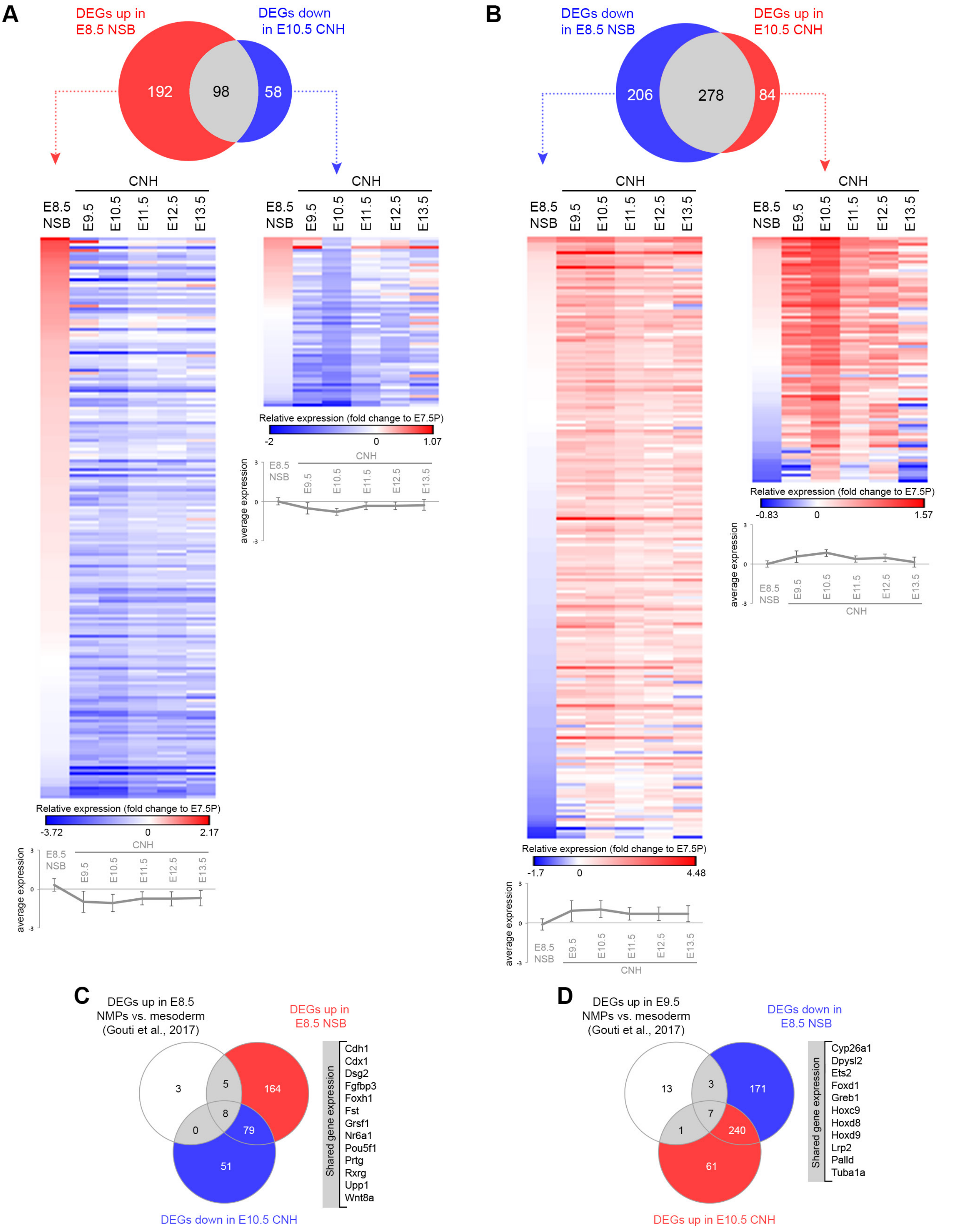


**Figure S5. Transcriptional changes at mid-trunk formation.**
(A) DEGs between the E8.5 NSB and the E10.5 CNH (intersections of these sets are shown in Fig. 5B). (B) Expression heatmaps of NMP-containing regions over time show a similar peak-decline pattern as those in Fig. 5Bb-c. The mean (± s.d.) is shown below each heatmap (see Table S5 for gene lists). (C-D) Up- or downregulated DEGs at E8.5 and E10.5, shown in A and B compared with enriched NMP genes (versus nascent mesoderm), obtained in a parallel single cell analysis (Gouti et al., 2017).

Development • Supplementary information

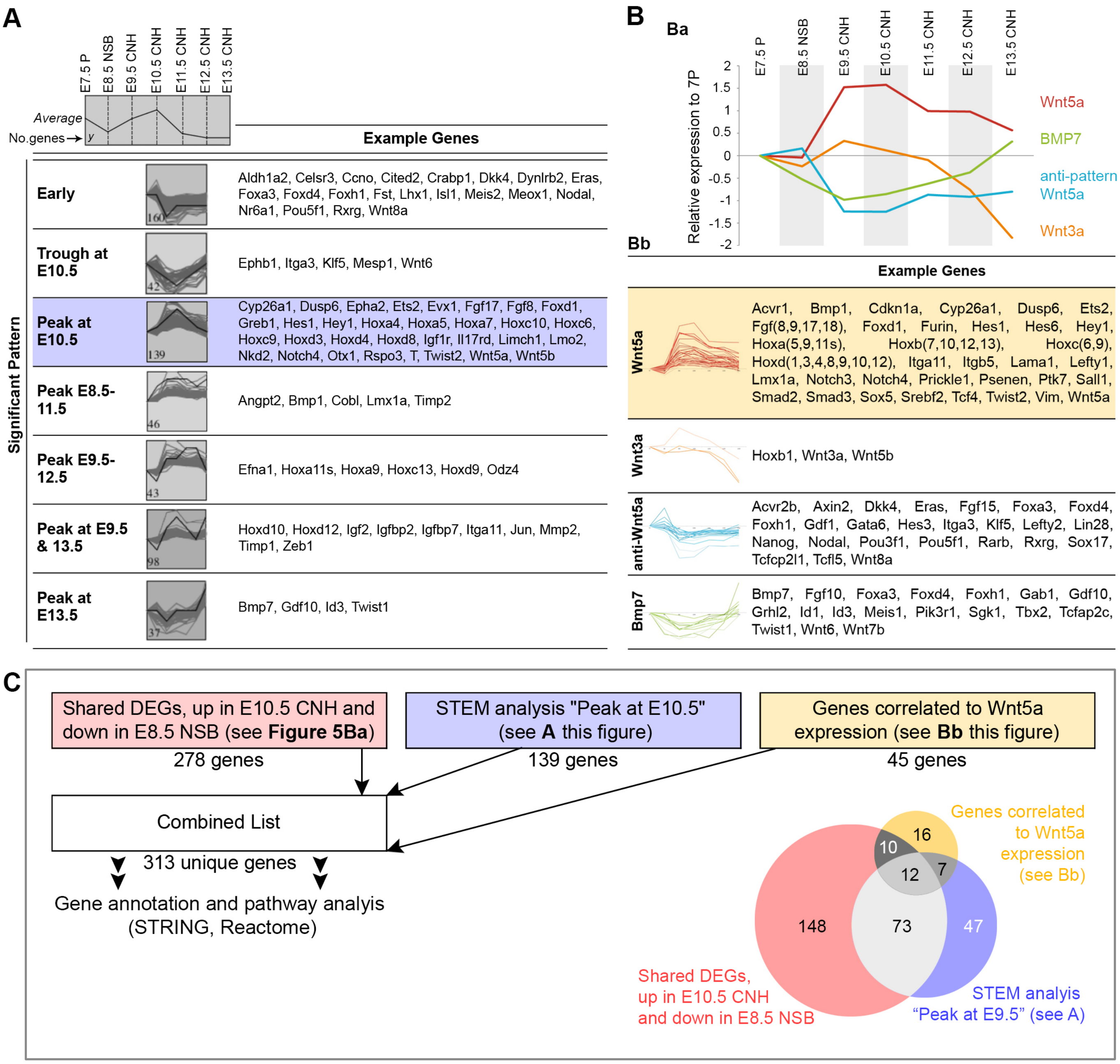


D

| GO-term | Description | p-value | KEGG ID | Description | p-value | Genes |
|---|---|---|---|---|---|---|
| GO.0009893 | positive regulation of metabolic process | 3.23E-05 | 4151 | PI3K-Akt signaling pathway | 0.00197 | Cdk6, Cdkn1a, Epha2, Fgf8/9/17/18, Ghr, Gng12, Igf1r, Insr, Itga11, Itgb5, Lama1, Vegfc |
| GO.0042127 | regulation of cell proliferation | 0.00953 | | | | |
| GO.0007155 | cell adhesion | 0.0146 | | | | |
| GO.0001568 | blood vessel development | 4.02E-06 | 4014 | Ras signaling pathway | 0.00616 | Epha2, Ets2, Fgf8/9/17/18, Gng12, Igf1r, Insr, Rasa3, Vegfc |
| GO.0043408 | regulation of MAPK cascade | 0.00797 | | | | |
| GO.0030334 | regulation of cell migration | 0.0399 | 650 | Butanoate metabolism | 0.0115 | Aacs, Abat, Acat2, Oxct1 |
| GO.0045785 | positive regulation of cell adhesion | 0.00508 | 4015 | Rap1 signaling pathway | 0.0402 | Epha2, Fgf8/9/17/18, Igf1r, Insr, Pard6g, Vegfc |
| GO.0050769 | positive regulation of neurogenesis | 0.0153 | | | | |
| GO.0048545 | response to steroid hormone | 0.0353 | 100 | Steroid biosynthesis | 0.0452 | Cyp51, Hsd17b7, Sc4mol |
| GO.1901342 | regulation of vasculature development | 0.00256 | | | | |
| GO.0001558 | regulation of cell growth | 0.0415 | | | | |
| GO.0016055 | Wnt signaling pathway | 0.0326 | | | | |
| GO.0007219 | Notch signaling pathway | 0.00427 | | | | |
| GO.0036342 | post-anal tail morphogenesis | 0.00773 | | | | |
| GO.0030947 | regulation of vascular endothelial growth factor receptor signaling pathway | 0.0199 | | | | |

**Figure S6. Upregulated genes at mid-trunk formation.**
(A) STEM analysis shows significant patterns in NMPs over time, relative to sample E7.5 P. Grey boxes show the number of genes in each pattern; the black line, their average (significant patterns defined as p≤0.05 by permutation test in STEM). (B) Correlated expression to selected genes: (Ba) chosen gene or pattern. (Bb) table of example genes that show a similar expression profile (Pearson's correlation factor ≥7). A full gene list can be found in Table S6. (C) A combined list of upregulated genes during mid-trunk formation, with the overlap showing in (D). Red, DEGs up in E10.5 CNH and down in E8.5 NSB. Blue, genes that peak at E10.5 (taken from the STEM analysis pattern in A). Yellow, genes with similar pattern to *Wnt5a* expression (taken from correlation analysis in Bb). (E) Overview of GO terms and KEGG signalling pathways in this combined list (see also Table S7).

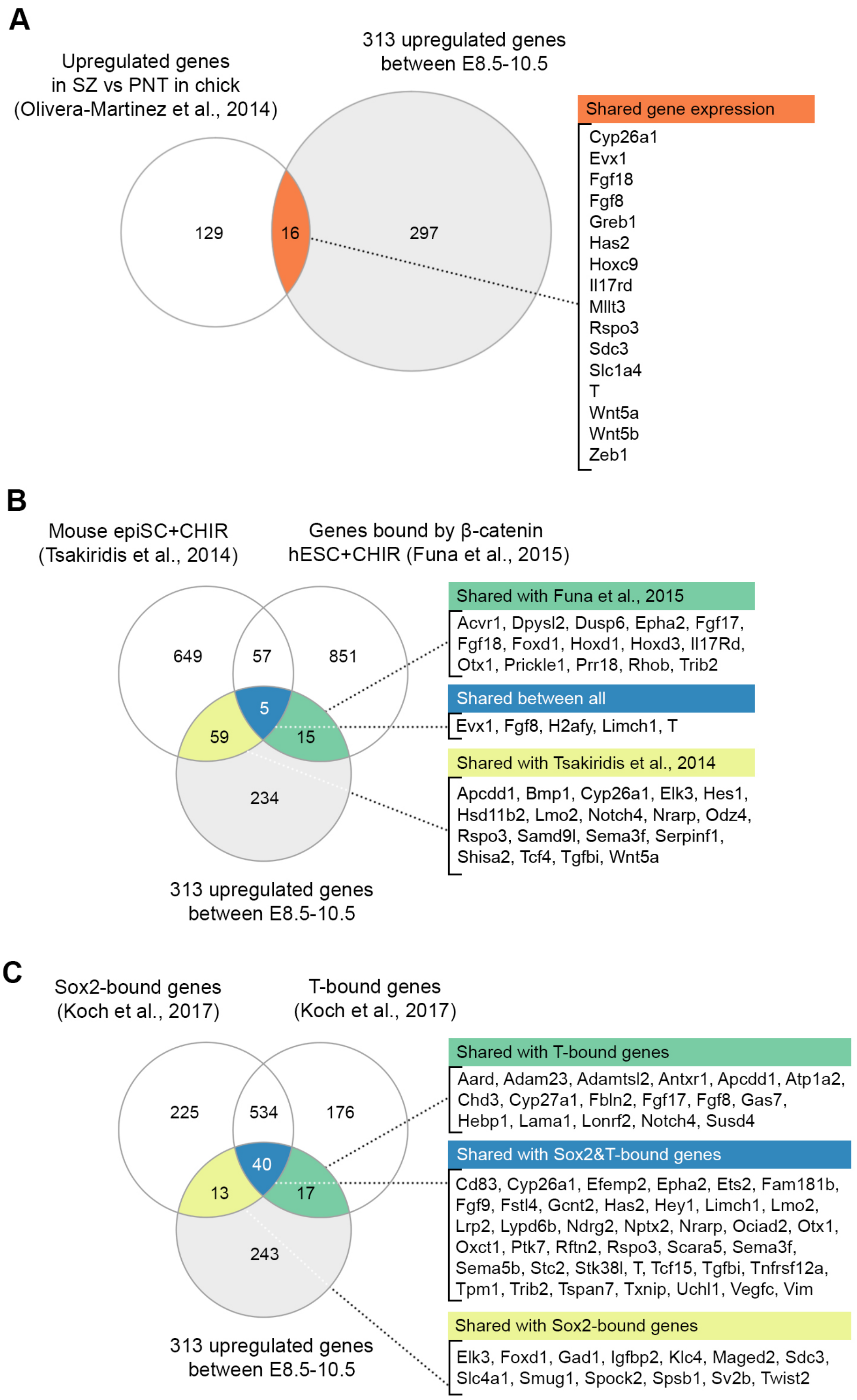


**Figure S7. Comparison of upregulated genes at mid-trunk formation to other available datasets.**
(A) Overlap between the 313 upregulated genes at mid-trunk formation with upregulated genes in the chick stem zone (versus the pre-neural tube) (Olivera-Martinez et al., 2014). (B) Overlap of 313-gene list with β-catenin bound genes in CHIR-treated human ESC (Funa et al., 2015) or mouse EpiSCs (Tsakiridis et al., 2014). (C) Overlap of 313-gene list with Sox2-, T- or Sox2&T- bound genes (Koch et al., 2017) (see also Table S7).

Development • Supplementary information

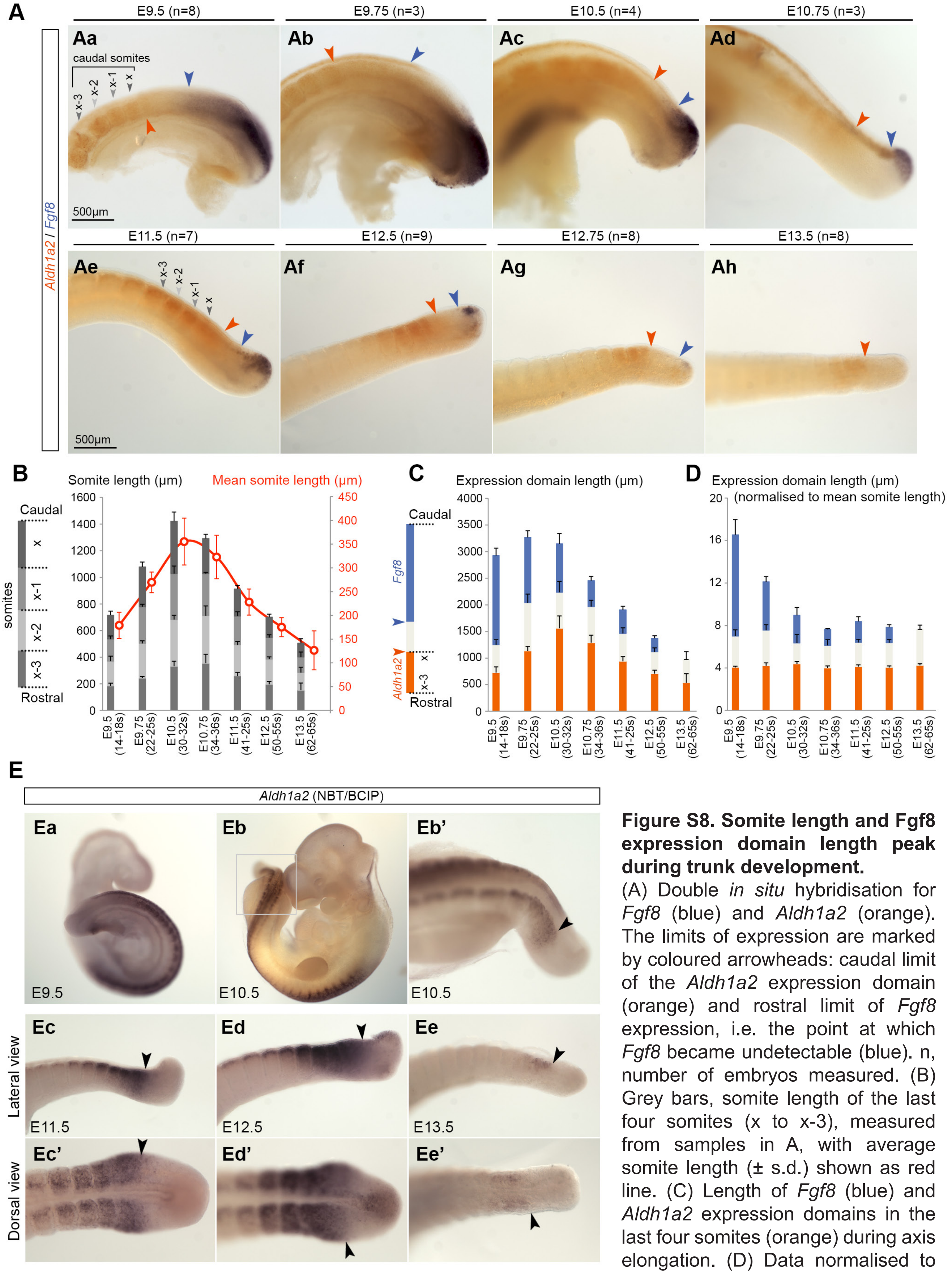


**Figure S8. Somite length and Fgf8 expression domain length peak during trunk development.** (A) Double *in situ* hybridisation for *Fgf8* (blue) and *Aldh1a2* (orange). The limits of expression are marked by coloured arrowheads: caudal limit of the *Aldh1a2* expression domain (orange) and rostral limit of *Fgf8* expression, i.e. the point at which *Fgf8* became undetectable (blue). n, number of embryos measured. (B) Grey bars, somite length of the last four somites (x to x-3), measured from samples in A, with average somite length (± s.d.) shown as red line. (C) Length of *Fgf8* (blue) and *Aldh1a2* expression domains in the last four somites (orange) during axis elongation. (D) Data normalised to the mean somite length. (E) Single *in situ* hybridisation controls for *Aldh1a2* transcripts at a series of developmental stages, using NBT/BCIP (5-bromo-4-chloro-3-indolyl-phosphate/nitro blue tetrazolium) instead of Fast Red as development substrate (used in A).

Development • Supplementary information

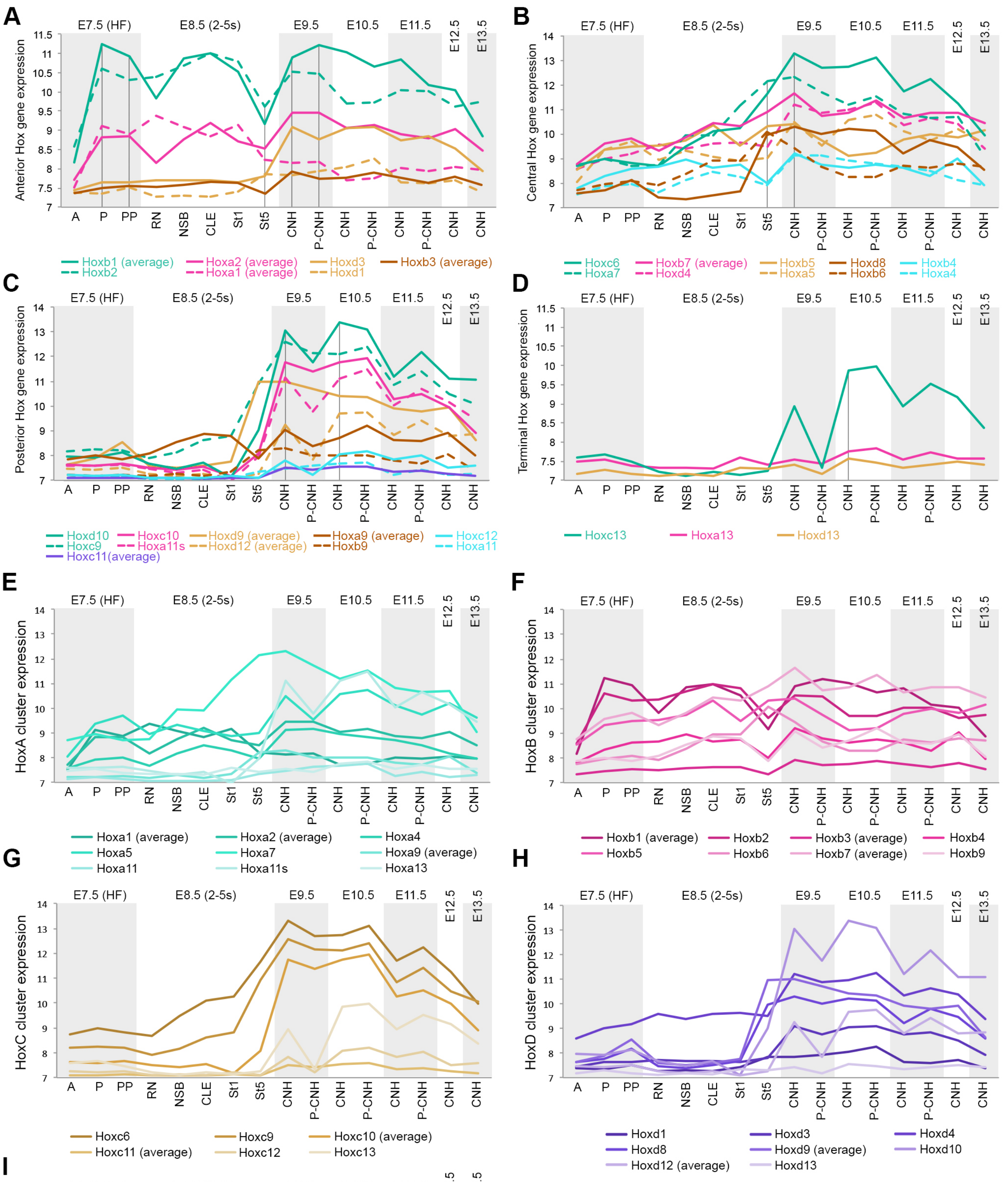


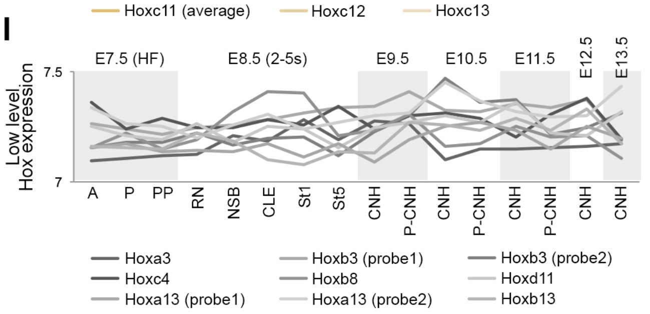


**Figure S9. Patterns in Hox gene expression.** *Hox* gene expression in all microarray samples, organised by paralogous groups (PG): PG1-3 (A), PG4-8 (B), PG9-12 (C) and PG13 (D). Multiple probes for the same gene were averaged. *Hox* expression organised by cluster: HoxA (E), HoxB (F), HoxC (G) and HoxD (H). *Hox* genes with low expression and no change between samples were excluded from the analysis (I).

Development • Supplementary information

**A**

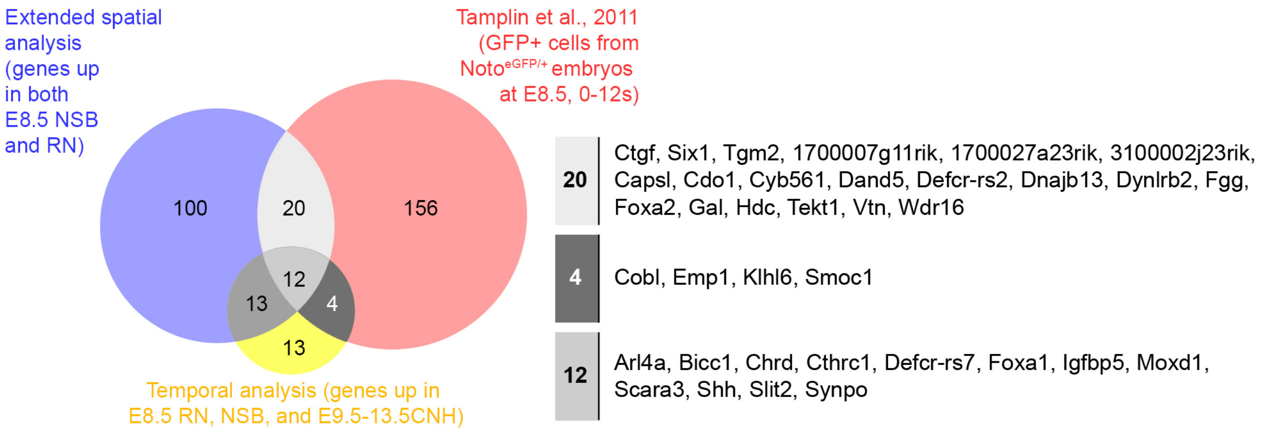


**B**

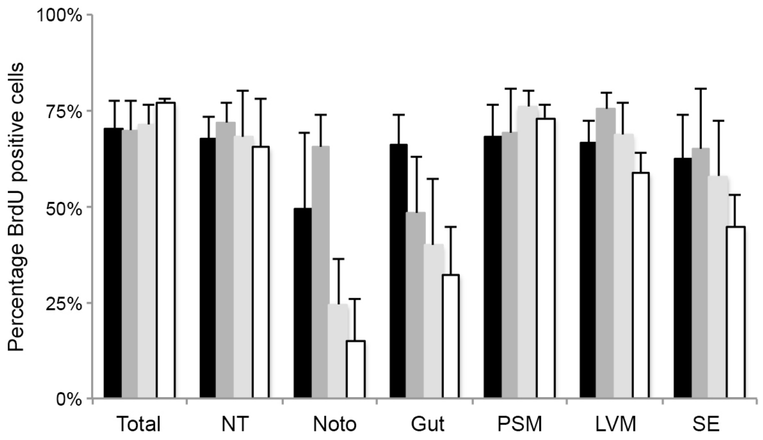


| | No. tails | No. TB sections | No. cells counted |
|---|---|---|---|
| ■ E10.5 | 5 | 12 | 16.831 |
| ■ E11.5 | 6 | 14 | 20.509 |
| ■ E12.5 | 6 | 18 | 31.717 |
| □ E13.5 | 4 | 5 | 17.102 |

**Percentage BrdU positive cells in tail bud tissues (mean ± s.d.)**

| | Total | NT | Noto | Gut | PSM | LVM | SE |
|---|---|---|---|---|---|---|---|
| **E10.5** | 70.4 ± 7.3 | 67.7 ± 5.5 | 49.6 ± 19.7 | 66.2 ± 7.8 | 67.9 ± 8.4 | 66.4 ± 5.8 | 62.7 ± 11.3 |
| **E11.5** | 69.9 ± 7.5 | 71.9 ± 5.4 | 65.5 ± 8.6 | 48.2 ± 14.9 | 69.2 ± 11.2 | 75.4 ± 4.0 | 65.0 ± 15.8 |
| **E12.5** | 71.4 ± 5.3 | 68.2 ± 11.8 | 24.5 ± 11.6 | 39.9 ± 17.5 | 76.1 ± 4.1 | 68.8 ± 8.1 | 58.0 ± 14.3 |
| **E13.5** | 76.9 ± 1.3 | 65.3 ± 12.6 | 15.1 ± 10.9 | 32.2 ± 12.5 | 73.1 ± 3.6 | 58.7 ± 5.3 | 44.5 ± 8.4 |

**Figure S10. Experimental controls and comparison of NotoP markers.**
(A) Comparison of E8.5 spatial analysis (DEGs shared in both RN and NSB), E8.5-13.5 temporal analysis (DEGs shared across E8.5 RN, NSB and E9.5-13.5 CNH samples) gene expression in sorted GFP$^{+}$ cells from E8.5 Noto$^{eGFP/+}$ embryos (0-12s) (Tamplin et al., 2011) (Table S8). (B) Percentage of BrdU$^{+}$ cells counted from immunostained tail sections after 4h *ex vivo* culture in the presence of BrdU. No. tails, no. tail bud (TB) sections and total no. cells counted as shown. Data is organised by tissue type: NT, neural tube, Noto, notochord; PSM, presomitic mesoderm; LVM, lateral and ventral mesoderm; SE, surface ectoderm.

Development • Supplementary information

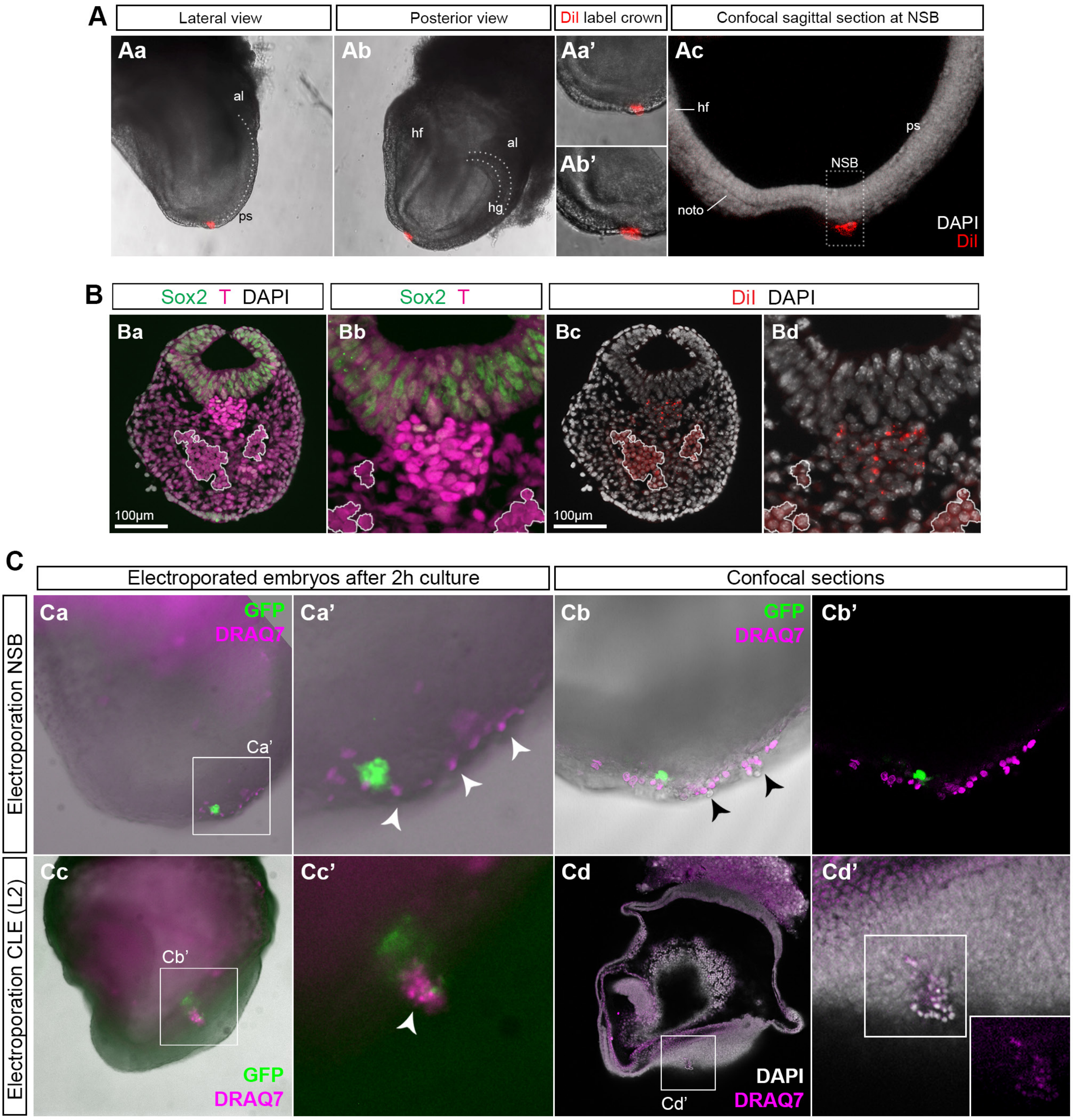


**Figure S11. DiI labelling and electroporation controls.**
(A) DiI labelling of the crown cells (red) in an E8.5 embryo: wholemount lateral (Aa), posterior view (Ab) validate the accuracy of initial labelling ($n_{embryos}$=3). (B) Sox2/T immunostained section of embryo labelled with DiI in the ventral node layer after 48h culture ($n_{embryos}$=2). White lines surround autofluorescent blood cells. (C) Cell death in NSB (Ca-Cb) or CLE (L2) (Cc-Cd) in electroporated embryos. After two hours *ex vivo* culture, $GFP^+$ cells (green) were observed at the electroporation site. Cell death, shown by DRAQ7 dye uptake (magenta), is located primarily at the ventral side of the embryo (arrowheads).

Development • Supplementary information

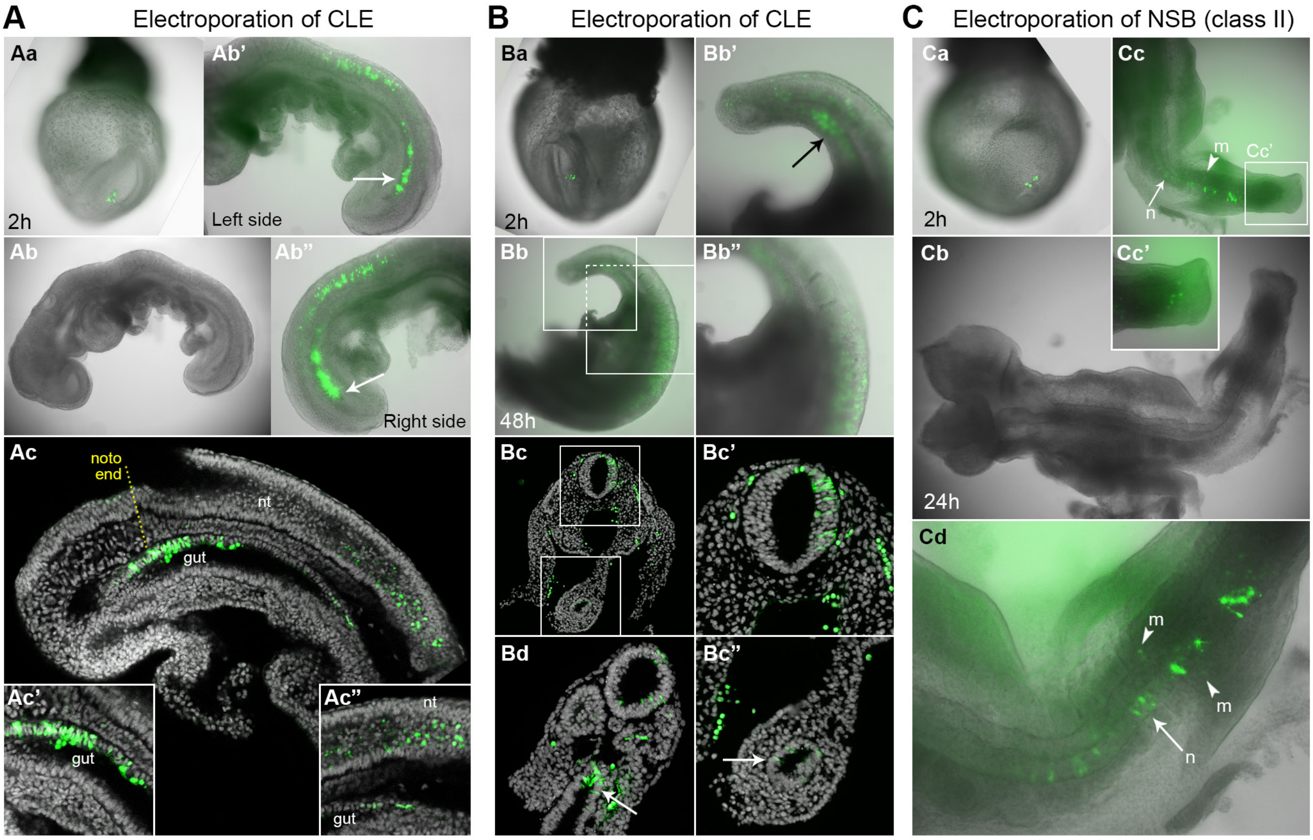


**D**

| Electroporation site | No. embryos labelled in Axis | No. embryos labelled in Tail bud | Total no. electroporated |
|---|---|---|---|
| PS | 2 | 2 | 2 |
| CLE | 10 | 2 | 10 |
| NSB | 14 | 6 | 20 |

**Figure S12. Fate of electroporated cells.**
Cell fate was examined in CLE-electroporated embryos after 24 (A) or 48h (B) *ex vivo* culture. In addition to the expected fate, the hindgut was labelled in all examined embryos (arrows; $n_{embryos}$=4), suggesting that also hindgut progenitors were electroporated. nt, neural tube. (C) Fate in a class II NSB-electroporated embryo. After 24h, GFP$^{+}$ cells were found in the neural tube (n) and paraxial mesoderm (m) of this embryo. (D) Contribution to the axial tissues and tail bud in electroporated samples.

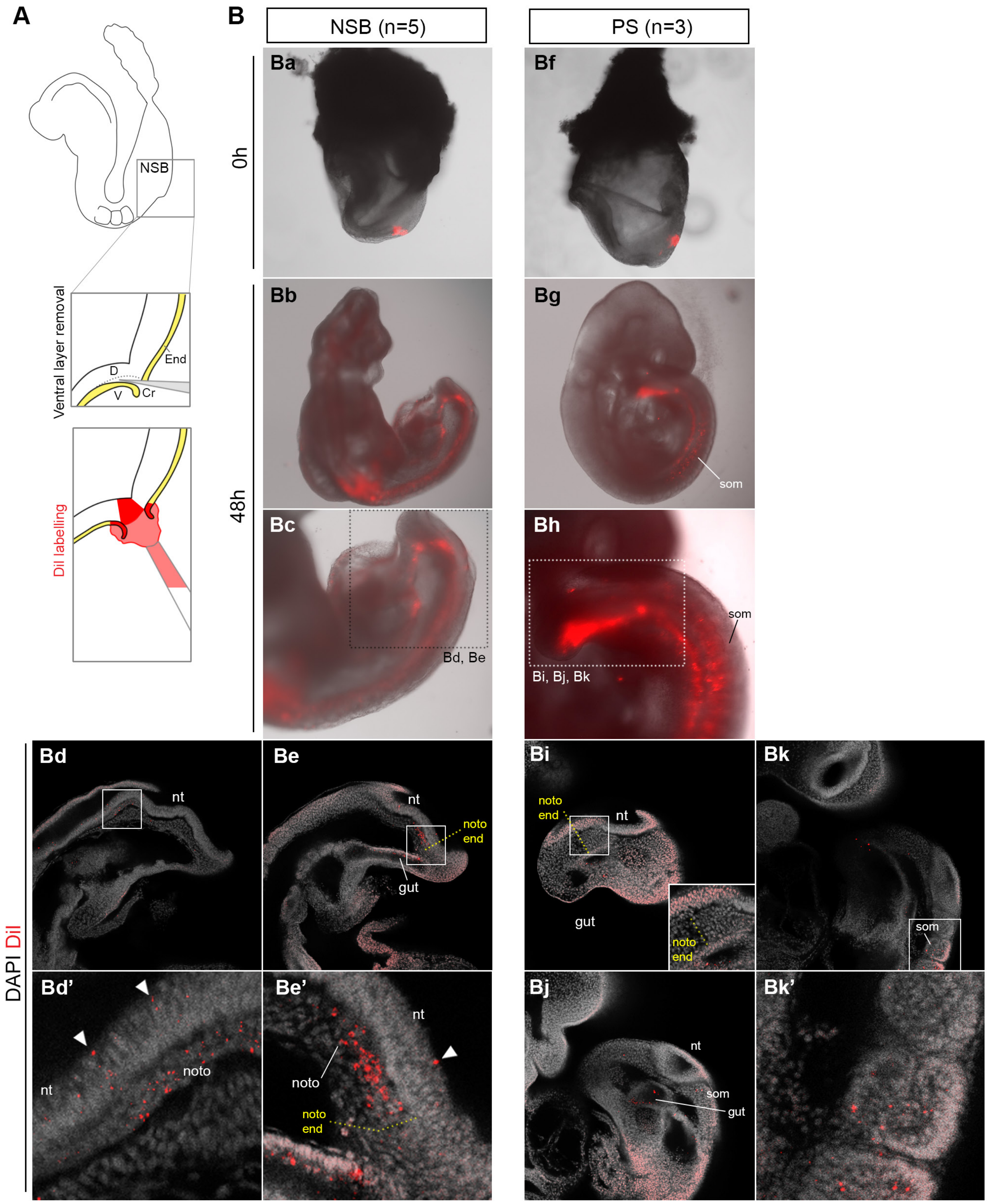


**Figure S13. Effect of ventral cell layer removal at the NSB or St3.**
(A) Experimental procedure schema illustrating the removal of the endoderm layer. A sharp glass needle was inserted posteriorly and pushed anteriorly to peel away the ventral endodermal cells. This ventral layer was further trimmed, after which DiI was poured on the damaged site, labelling the cell layer above as well as the endodermal cells. (B) Fate and phenotype after ventral layer removal in the NSB (Ba-Be) or St3 (Bf-Bk) after 48h *ex vivo* culture. (Bb-Bc) Removal of the ventral node layer resulted in abnormal growth (similar to class II phenotypes in electroporation, $n_{embryos}$=5, Fig. 8C). DiI-labelled cells were found in the notochord and neural tube (Bd-Be). Control embryos grew normally (n=3) and DiI was found in somite and gut tissues (Bg-Bh). Red dots, DiI (pink, background due to high exposure). nt, neural tube; noto, notochord; som, somites; white arrowheads, DiI label in ventral neural tube.

Development • Supplementary information

## SUPPLEMENTARY TABLES

**Table S1. Full list of DEGs in the primitive streak and St5 (supplementary to Figure 2).**
(Tab 1) Unique DEGs for St5, not shown in Figure 2A. (Tab 2) List of unique DEGs for each region at E8.5. Columns allow for pairwise comparison between two regions (1, ≥1.5 fold upregulated; -1, ≥1.5 fold downregulated; 0, no significant change).

Click here to download Table S1

**Table S2. Hierarchical clustering of DEGs ≥1.5 fold change across the primitive streak (supplementary to Figure S3).**
Tab 1 shows all DEGs ≥1.5 fold changed across the E8.5 sample set that were used in hierarchical clustering of Figure S3 and analysed for GO-terms/KEGG pathways. Three major clusters were identified and several sub-clusters could be distinguished. Subsequent tabs show all GO-terms and KEGG pathways for each pattern obtained from the STRING online database (accessed 10th April 2017).

Click here to download Table S2

**Table S3. STEM analysis across different sample sets (supplementary to Figure 4 and S6).**
STEM analysis shows significant patterns in two datasets: (1) in the E7.5 embryo (A, P and PP samples) and (2) in comparable NMP-containing regions (E8.5 NSB to E9.5-E13.5 CNH, normalised to E7.5 P). Tables shows significant patterns, and the genes and enriched GO-terms within each pattern ($p \leq 0.05$, defined by permutation test in STEM).

Click here to download Table S3

**Table S4. DEGs unique to and shared between LPMPs at E7.5/8.5 (supplementary to Figure 4).**
Full list of DEGs not shown in Figure 4C.

Click here to download Table S4

**Table S5. Change in NMP gene expression during axis elongation (supplementary to Figure 5 and Figure S5).**
Lists of DEGs not shown in Figure 5B (Tab 1) and 5C (Tab 2). (Tab 3) Shared gene expression between DEGs uniquely changed at E8.5, E10.5 and DEGs upregulated in E8.5 or E9.5 single NMPs (compared to mesoderm) (Gouti et al., 2017).

Click here to download Table S5

**Table S6. Correlated gene expression to selected genes or expression pattern (supplementary to Figure S6).**
Genes that correlate to the selected gene or profile in NMP-containing regions during axis elongation (E8.5 NSB, E9.5-E13.5 CNH). Selected genes are *Bmp1, Bmp7, Wnt3a, Wnt5a*, and anti-correlated pattern to *Wnt5a*. Right column, genes with a similar pattern to the selected gene (Pearson's correlation factor ≥0.7). Left, curated genes and their temporal expression (≥1.5 fold change, normalised to 7P expression).

Click here to download Table S6

**Table S7. Genes upregulated between E8.5 and E10.5 NMPs: construction of list, its annotation and comparison to other datasets (supplementary to Figure S6 and Figure S7).**
(Tab 1) Genes upregulated during mid-trunk formation in NMPs, with the overlap between different analyses shown. Red, DEGs that were upregulated in the E10.5 CNH, and down at E8.5 NSB. Blue, genes that peak at E9.5 (from STEM analysis). Yellow, genes with similar temporal expression pattern to *Wnt5a*. (Tab 2) Manual annotation of the combined list shown in Tab 1. STRING annotation (Tab 3) and GO-terms associated with the combined list (Tab 4). Tab 5: Comparison with Olivera-Martinez et al., 2014. Tab 6: Comparison with Funa et al., 2015 and Tsakiridis et al., 2014. Tab 7: Comparison with Koch et al., 2017.

Click here to download Table S7

**Table S8. Markers of notochord and node during axis elongation (supplementary to Figure S10).**
(Tab 1) Common gene expression in E8.5 RN, NSB and E9.5 to E13.5 CNH versus all other samples (dataset normalised to E7.5P). Data is separated into known and potential novel markers for the node/notochord. Red: averaged expression from different probes on the microarray. (Tab 2) Comparison between Tamplin et al., 2011. and samples containing NotoPs: enriched in E8.5 RN and NSB versus E8.5 samples (‘spatial analysis’), and in E8.5 RN, NSB and E9.5-13.5 CNH (‘temporal analysis’, see Tab 1).

Click here to download Table S8

Development • Supplementary information

| | Embryo | Mouse line | Plasmid | GFP location at 2h | Culture period | Phenotype class |
|---|---|---|---|---|---|---|
| CLE | 1 | MF1 | pCAG-GFP | L1 | 24h | I |
| | 2 | MF1 | pCAG-Cre:GFP | L1 | 24h | N |
| | 3 | MF1 | pCAG-GFP | L1 | 24h | N |
| | 4 | sGFP | pCAG-Cre:GFP | L1 | 48h | N |
| | 5 | sGFP | pCAG-Cre:GFP | L1 | 48h | N |
| | 6 | sGFP | pCAG-Cre:GFP | LL1 | 48h | N |
| | 7 | sGFP | pCAG-Cre:GFP | L1 | 48h | N |
| | 8 | sGFP | pCAG-Cre:GFP | L1 | 48h | N |
| | 9 | sGFP | pCAG-Cre:GFP | L1 | 48h | N |
| | 10 | sGFP | pCAG-Cre:GFP | L1 | 48h | N |
| PS | 11 | sGFP | pCAG-Cre:GFP | St1 | 48h | N |
| | 12 | MF1 | pCAG-GFP | St3 | 24h | N |
| NSB | 13 | sGFPxMF1 | pCAG-Cre:GFP | RN+NSB | 24h | I |
| | 14 | sGFPxMF1 | pCAG-Cre:GFP | NSB | 24h | I |
| | 15 | MF1 | pCAG-GFP | NSB | 24h | II |
| | 16 | MF1 | pCAG-GFP | NSB | 24h | II |
| | 17 | MF1 | pCAG-GFP | NSB | 48h | II |
| | 18 | MF1 | pCAG-GFP | NSB | 24h | II |
| | 19 | MF1 | pCAG-GFP | NSB | 24h | II |
| | 20 | MF1 | pCAG-GFP | NSB | 24h | II |
| | 21 | MF1 | pCAG-GFP | NSB | 24h | II |
| | 22 | MF1 | pCAG-GFP | NSB | 24h | I |
| | 23 | MF1 | pCAG-GFP | NSB | 24h | I |
| | 24 | MF1 | pCAG-GFP | NSB | 24h | I |
| | 25 | MF1 | pCAG-GFP | NSB | 48h | N |
| | 26 | MF1 | pCAG-GFP | NSB | 48h | II |
| | 27 | MF1 | pCAG-GFP | NSB | 48h | II |
| | 28 | MF1 | pCAG-GFP | RN+NSB | 24h | I |
| | 29 | MF1 | pCAG-GFP | NSB | 24h | II |
| | 30 | MF1 | pCAG-GFP | NSB | 24h | II |
| | 31 | MF1 | pCAG-GFP | NSB | 24h | II |
| | 32 | MF1 | pCAG-GFP | NSB | 24h | II |
| Control | 33 | MF1 | pCAG-GFP | EP control | 24h | N |
| | 34 | MF1 | pCAG-GFP | EP control | 24h | N |
| | 35 | MF1 | pCAG-GFP | EP control | 24h | N |
| | 36 | MF1 | pCAG-GFP | EP control | 24h | II |

**Table S9. Overview of electroporated embryos.**
Overview of electroporated embryos of CLE, primitive streak or NSB-targeted regions in E8.5 (2-5s) embryos. Table shows embryo number, corresponding genotype, plasmid and culture period. After 24/48h *ex vivo* development all embryos were assessed on their developmental features and assigned a phenotype class: N, normal; I, class I; II, class II.

| Real-time qPCR primer list | | |
|---|---|---|
| Gene | | Primer sequence |
| Bmp4 | Fwd | caacccaattatgggctggc |
| | Rev | ccacaatccaatcattccagc |
| Evx1 | Fwd | gtttcaagaccgcggagat |
| | Rev | tgacgcttgtccttcatgc |
| Fgf4 | Fwd | ccggttcttcgtggctatga |
| | Rev | cttactgagggccatgaacatacc |
| Fgf8 | Fwd | atggcagaagacggagacc |
| | Rev | ttgttcatgcagatgtagagacc |
| Foxa2 | Fwd | catccgactggagcagcta |
| | Rev | gcgcccacataggatgac |
| Mixl1 | Fwd | agttgctggagctcgtcttc |
| | Rev | agggcaatggaggaaaactc |
| Sox2 | Fwd | ggcggcaaccagaagaacag |
| | Rev | gcttggcctcgtcgatgaac |
| T(Bra) | Fwd | ccaaggacagagagacggct |
| | Rev | agtaggcatgttccaagggc |
| TBP | Fwd | ggggagctgtgatgtgaagt |
| | Rev | ccaggaaataattctggctca |
| Wnt3a | Fwd | aatggtctctcgggagtttg |
| | Rev | cttgaggtgcatgtgactgg |

**Table S10. Real-time qPCR primer list.** Overview of the primers used in Fig. S1B.

Development • Supplementary information
